# PLXFPred: interpretable cross-attention networks with hierarchical fusion of multi-modal features for predicting protein–ligand interactions and affinities

**DOI:** 10.1093/bioinformatics/btaf662

**Published:** 2026-01-09

**Authors:** Jixiang Li, Ruilin Cai, Ziteng Wang, Ye Sun, Wenge Yang, Yonghong Hu

**Affiliations:** College of Biotechnology and Pharmaceutical Engineering, Nanjing Tech University, Nanjing 211816, P. R. China; State Key Laboratory of Materials-Oriented Chemical Engineering, Nanjing Tech University, Nanjing 211816, P. R. China; College of Biotechnology and Pharmaceutical Engineering, Nanjing Tech University, Nanjing 211816, P. R. China; State Key Laboratory of Materials-Oriented Chemical Engineering, Nanjing Tech University, Nanjing 211816, P. R. China; College of Biotechnology and Pharmaceutical Engineering, Nanjing Tech University, Nanjing 211816, P. R. China; State Key Laboratory of Materials-Oriented Chemical Engineering, Nanjing Tech University, Nanjing 211816, P. R. China; State Key Laboratory of Materials-Oriented Chemical Engineering, Nanjing Tech University, Nanjing 211816, P. R. China; College of Food Science and Light Industry, Nanjing Tech University, Nanjing 211816, P. R. China; College of Food Science and Light Industry, Nanjing Tech University, Nanjing 211816, P. R. China; School of Pharmaceutical Sciences, Nanjing Tech University, Nanjing 211816, P. R. China; State Key Laboratory of Materials-Oriented Chemical Engineering, Nanjing Tech University, Nanjing 211816, P. R. China; College of Food Science and Light Industry, Nanjing Tech University, Nanjing 211816, P. R. China

## Abstract

**Summary:**

Accurately predicting protein–ligand interactions and binding affinities is essential for advancing structural biology. Despite recent advancements in deep learning, achieving rapid and precise predictions remains a challenging task. Our approach, Protein–Ligand Cross-Modal Fusion Predictor (PLXFPred), extracts physicochemical properties from amino acid sequences and SMILES. Additionally, it leverages pre-trained models to derive high-dimensional features. GATv2 and BILSTM were used to process the structural and sequence features, respectively. The model’s core involves fusing sequence and graph features via a cross-modal cross-attention mechanism, followed by a multi-modal hierarchical fusion strategy that integrates high-level graph, early fusion, and cross-fusion features. Residual connections and conditional domain adversarial learning improve generalization to previously unseen protein–ligand pairs. Compared to state-of-the-art models, PLXFPred demonstrates superior performance, reducing errors (RMSD, MAE, SD) by over 50%, while providing interpretable biological insights through attention weight visualization and SHAP analysis.

**Availability and implementation:**

The resource codes are available at https://github.com/xiyuyangtuo/PLXFPred/.

## 1 Introduction

Accurately predicting protein–ligand binding and affinity is crucial for understanding biomolecular functions, designing new drugs, and optimizing drug efficacy (Clark *et al.* 2021, Volkov *et al.* 2022). Traditional methods like molecular dynamics (Wei *et al.* 2022), crystallography (Carlucci *et al.* 2023, Shen *et al.* 2025), and NMR (Liu *et al.* 2023, Nepravishta *et al.* 2024) provide insights but are computationally costly, especially for large complexes (Wang *et al.* 2020, Wang *et al.* 2021a,b). Molecular docking is efficient but less accurate (Du *et al.* 2023). Traditional machine learning improves cost and accuracy but struggles with nonlinear, high-dimensional data (Gönen 2012, Shen *et al.* 2021a,b) and lacks generalization and interpretability (Öztürk *et al.* 2018, Shen *et al.* 2021a,b). Recent advances in deep learning identify complex patterns, yet precise binding affinity prediction remains challenging, as most studies focus on binary classification (Nag *et al.* 2022, Isert *et al.* 2023).

Current deep learning methods for protein–ligand interactions and affinity prediction fall into two categories: sequence-based and structure-based.

Sequence-based models, like DeepDTA (Öztürk *et al.* 2018) and deepLIP, use 1D-CNNs (Yang *et al.* 2024) on protein and ligand sequences or pocket-ligand interactions. Structure-based deep learning methods use 3D structural data to enhance protein–ligand affinity prediction. GraphscoreDTA utilizes GNNs with bidirectional transfer [18], whereas Mastropietro *et al.* use GNN variants (Mastropietro *et al.* 2023). 3D-CNNs process molecular surfaces, and BAP uses inter-molecular contact profiles. Additionally, 3D-CNNs effectively capture spatial interaction patterns (Ragoza *et al.* 2017, Liu *et al.* 2021, Li *et al.* 2023), such as BAP, using inter-molecular contact profiles (IMCPs) (Wang and Chan 2022). Graph-Transformer models non-covalent interactions for better generalization (Lai *et al.* 2024). However, these methods rely on costly, hard-to-obtain 3D structural data, which can be inaccurate (Voitsitskyi *et al.* 2023). Also in the structure are methods based on protein–ligand complexes (Seo *et al.* 2021), which require not only structure but also higher precision (Tan *et al.* 2023). As a result, sequence-based methods are preferred due to the easier availability of sequence data, reducing costs and minimizing computational errors.

Single-feature models struggle with accurately capturing protein–ligand interactions due to complex non-covalent forces and 3D spatial alignments. For example, while conventional models using CNN or GNN perform well on specific benchmarks, their accuracy and generalization decline significantly when applied to novel proteins or those markedly different from the training data (Hong *et al.* 2024). To address this, multi-modal frameworks like DrugLAMP combine molecular graphs and protein sequences using pre-trained language models (PLMs) and attention mechanisms, combining pocket-guided and paired multi-modal attention to better elucidate ligand-protein interactions (Luo *et al.* 2024, Zhao *et al.* 2024a,b). Similarly, NHGNN-DTA merges BILSTM (Bi-directional Long Short-Term Memory) with Graph Isomorphism Networks for improved ligand-protein prediction (He *et al.* 2023). Despite progress, challenges in accuracy, generalization, and interpretability remain.

Building on these studies, we decode and extract protein–ligand interaction patterns solely from sequence data to develop the Protein–Ligand Cross-Modal Fusion Predictor (PLXFPred) model. A graph attention network is used to capture local features, the BILSTM and Transformer architectures are used to analyze global features, and features from different periods are fused through a cross-attention mechanism (Liu and Guo 2019, Xu *et al.* 2023). Additionally, an adversarial training mechanism enhances the model’s generalization and robustness, enabling better adaptation to data across diverse domains.

## 2 Materials and methods

### 2.1 PLXFPred architecture

**Figure 1.**
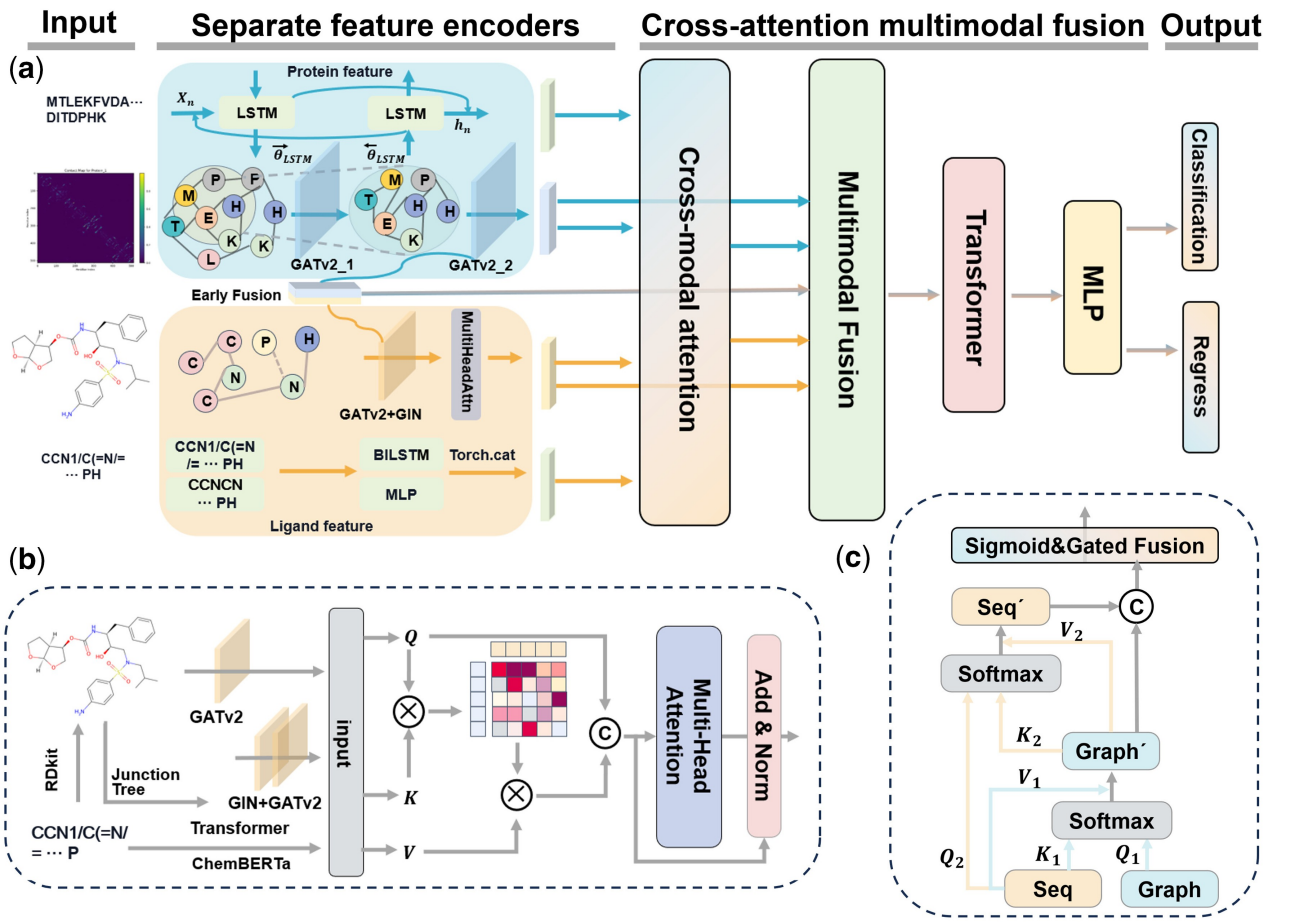
Overview of the PLXFPred framework. (a) PLXFPred processes protein and ligand inputs using a GAT to encode graph features and a BILSTM network for sequence features. These features are fused via cross-attention, integrating graph and sequence data, while early fusion combines protein and ligand graph features. The resulting early fusion, original, and cross-fusion features are merged into multi-modal features. A Transformer layer with self-attention refines these features, and residual connections ensure stable gradient flow. Outputs for classification and regression are generated via a MLP layer. (b) In ligand graph encoding, the SMILES sequence is converted to a molecular graph using RDKit and encoded with GATv2. Simultaneously, tree decomposition breaks the graph into cluster features, encoded first by GIN and then by GATv2. Pre-trained ChemBERTa features are processed through a Transformer layer, and all three feature sets are fused via self-attention to create a comprehensive ligand graph representation. (c) The cross-attention module integrates sequence features into the graph, then incorporates graph features back into the sequence, merging the updated features. Gated fusion controls the balance of graph and sequence contributions in the final output.

The deep learning architecture of PLXFPred is illustrated in Fig. 1a, which utilizes a cross-attention mechanism to integrate the features of proteins and ligands at different time points and predicts the results after multimodal fusion. The model primarily leverages GATs with multi-scale pooling to capture intricate molecular topological structures from the local information of proteins and ligands, while sequence data is processed using BILSTM and Transformer-based mechanisms. As shown in Fig. 1b, ligand graph processing is divided into three components: the molecular graph is encoded by a single-layer GATv2, while the cluster features, decomposed into a tree structure, are first processed by a GIN layer and then by a GATv2 layer. Additionally, ChemBERTa, a pre-trained model, utilizes a Transformer layer with multi-head attention to extract key characteristics, which are subsequently integrated with the other features via self-attention (Kang *et al.* 2022). Cross-attention fusion of graph features and sequence features is illustrated in Fig. 1c, with dynamic adjustment of their contributions facilitated through a gated fusion mechanism. The final multi-modal fusion combines feature representations from different periods, utilizing Transformer layers and residual connections to assign weights and model global interactions dynamically. The resulting features are fed into a parallel MLP with two branches: a regression branch for predicting affinity values and a classification branch for binary classification.

### 2.2 ProteinGraphEncoder

The ProteinGraphEncoder constructs initial node representations from multi-modal protein features using position encoding, a Transformer for evolutionary features, and an MLP for sequence and property encoding (Formula 1). A two-layer enhanced GATv2 convolution computes dynamic attention weights with edge features, stabilized by LeakyReLU, softmax, GraphNorm, and ELU (Formulas 2–3) (Chang and Liu 2025). The second layer integrates residual connections to preserve information (Formula 4), with dropout enhancing sparsity. Finally, Triple pooling (attention, maximum, and average) yields a comprehensive representation (Formula 5).


(1) $$h_{i}^{0}(\mathrm{pos}\_\mathrm{emb}(p_{i});\mathrm{Transformer}(W_{e}{\mathrm{evo}}_{i});W_{oh}one\_\mathrm{hot};W_{aa}W_{i})$$

(2) $$\alpha_{ij}^{1}=\mathrm{softmax}(a^{T}L\mathrm{eakyRelU}(W_{q}h_{i}^{0}+W_{k}h_{j}^{0}+W_{e}e_{ij}))$$

(3) $$h_{i}^{1}=\mathrm{ELU}(\mathrm{GraphNorm}(\sum_{j\in N(i)}\alpha_{ij}^{1}W_{v}h_{j}^{0}))$$

(4) $$h_{i}^{2}=\mathrm{ELU}(\mathrm{GraphNorm}(\mathrm{GATv}2(h^{1})+W_{\mathrm{res}}h_{i}^{0})$$

(5) $$h_{\mathrm{graph}}=W_{p}[\sum_{i\in g}\alpha_{i}h_{i}^{2};\max_{i\in g}h_{i}^{2};\frac{1}{\left|g\right|}\sum_{i\in g}h_{i}^{2}]$$

### 2.3 LigandGraphEncoder

The LigandGraphEncoder processes ligand graphs in three stages. First, GATv2Conv updates atomic features with attention-weighted neighbor aggregation, stabilized by residual connections, graph normalization, and ELU (Formula 6). Next, GINConv and GATv2Conv capture clique and enhance the representation by capturing higher-order molecular patterns through structural context (Formulas 7–8) (Chang and Liu 2025). ChemBERTa features are enriched via the Transformer and fused with graph features (Formula 9) (Wang *et al.* 2024, Zhang *et al.* 2025). Finally, Formula 10 uses self-attention to compute inter-modal similarity across molecular graph, cluster graph, and ChemBERTa features, dynamically weighting them to produce fused representations (Zhao *et al.* 2024a,b). Formula 11 adds residual connections to preserve original feature information and prevent gradient vanishing.


(6) $$h_{\mathrm{mol}}^{1}=\mathrm{ELU}(\mathrm{GraphNorm}(\sum_{j\in N(i)}\alpha_{ij}W_{v}h_{j}^{0})+W_{\mathrm{res}}h_{i}^{0})$$

(7) $$h_{\mathrm{clique}}^{1}=\mathrm{GIN}(h_{\mathrm{clique}}^{0},\varepsilon_{\mathrm{cross}})$$

(8) $$h_{\mathrm{clique}}^{2}=\mathrm{ELU}(\mathrm{GraphNorm}(\mathrm{GATv}2(h_{\mathrm{clique}}^{1}))+W_{\mathrm{res}}^{\mathrm{clique}}h_{\mathrm{clique}}^{0})$$

(9) $$h_{\mathrm{chem}}^{1}=\mathrm{Transformer}(W_{c}^{\mathrm{proj}}\cdot \mathrm{ChemBERTa}(\mathrm{SMILES}))$$

(10) $$\mathrm{Attn}(Q,K,V)=\mathrm{softmax}(\frac{(W_{d}H_{\mathrm{stack}}){\left(W_{k}H_{\mathrm{stack}}\right)}^{T}}{\sqrt{d}})(W_{v}H_{\mathrm{stack}});\;H_{\mathrm{stack}}=[\begin{array}{c} h_{\mathrm{mol}}^{\mathrm{pool}}\\ h_{\mathrm{clique}}^{\mathrm{pool}}\\ h_{\mathrm{chem}} \end{array}]\;\in \;R^{3\times d}$$

(11) $$h_{\mathrm{final}}=BN(W_{f}c\mathrm{oncat}[\mathrm{Attn}(Q,K,V),\frac{1}{3}\sum_{i=1}^{3}h_{i}])$$

### 2.4 SequenceEncoder (ProteinSequenceEncoder and LigandSequenceEncoder)

The ProteinSequenceEncoder processes protein sequences using two BILSTM layers to extract global and contextual features, with residual connections preventing information loss (Formula 12). A multi-head self-attention mechanism highlights key residues, and adaptive maximum pooling condenses features into a fixed dimension for downstream tasks (Formula 13). Ligand sequences are processed similarly, integrating SMILES and atomic features via a feedforward network, BILSTM, and adaptive pooling, with residual connections preserving SMILES information (Formulas 14–15).


(12) $$h_{2}={\mathrm{BiLSTM}}_{2}({\mathrm{BiLSTM}}_{1}(x_{\mathrm{protein}}))+W_{\mathrm{res}}{\mathrm{BiLSTM}}_{1}(x_{\mathrm{protein}})$$

(13) $$h_{\mathrm{final}}=\mathrm{Maxpool}(\mathrm{Attn}(Q,K,V))\;\in \;R^{d}$$

(14) {hmol=Maxpool(MLP(smiles)_atmoic)) ∈ Rdhsmiles=Maxpool(BiLSTM(mol_x_feat)) ∈ Rd

(15) $$h_{\mathrm{final}}=W_{\mathrm{out}}(|h_{\mathrm{smiles}}||h_{\mathrm{mol}}|+W_{\mathrm{res}}h_{\mathrm{smiles}})\;\in \;R^{d}$$

### 2.5 Cross-modal attention

This module integrates graph and sequence features using multi-head attention (MHA), with graph features serving as the Query and sequence features as the Key/Value (Formula 16). Layer normalization, residual connections, and an FFN enhance stability and expressiveness (Formula 17). Gated fusion, using a Sigmoid-activated layer, dynamically merges refined features for a high-quality representation (Formula 18).


(16) {gx′=Norm(gx+MHA(gx,sx,sx))sx′=Norm(sx+MHA(sx,gx,gx))

(17) {gx″=Norm1g(gx′+FFN(gx′)+gxres)sx″=Norm1s(sx′+FFN(sx′)+sxres))

(18) $$f_{\mathrm{output}}=\mathrm{Sigmoid}(W[g_{x}^{\prime \prime };s_{x}^{\prime \prime }])⨀g_{x}+(1S\mathrm{igmoid}(W[g_{x}^{\prime \prime };s_{x}^{\prime \prime }]))⨀s_{x}^{\prime \prime }$$

### 2.6 Multi-modal fusion

FusedAffinityPredictor is a multi-modal fusion model tailored for predicting binding affinity and classifying activity in protein–ligand interactions. It uses a hierarchical fusion strategy: early fusion (Formula 19) uses multi-head attention to capture local atom-level interactions. Mid-level fusion (Formula 20) applies a cross-transformer to integrate and denoise ligand sequence features. Subsequently, all features—including original graph features, cross-modal fusion outputs, and early interaction features—are combined and processed by a Transformer fusion layer with residual connections to capture complex relationships (Formula 21). Finally, the fused features are passed to three parallel MLPs (Formula 22) for affinity regression, activity classification, and domain adaptation via a gradient reversal layer.


(19) $$h_{\mathrm{early}}=\mathrm{MultiHeadAttention}(\mathrm{Pool}(h_{\mathrm{pro}\_\mathrm{raw}});\mathrm{Pool}(h_{\mathrm{lig}\_\mathrm{raw}}))$$

(20) {hpro_fused=BalancedCrossTransformer(hpro_graph,hpro_seq)hlig_fused=BalancedCrossTransformer(hlig_graph,hlig_seq)

(21) $$f_{\mathrm{fused}}=\mathrm{Transformer}(W_{1}f_{\mathrm{combined}})+W_{2}f_{\mathrm{combined}}$$

(22) {yreg=RegressorHead(ffused)ycls=Sigmoid(ClassifierHead(ffused))ydom=softmax(DomainHead(GRL(ffused)))

### 2.7 Dataset

We used the PDBBind v2020, CASF-2016, CASF-2013, and Human datasets (Liu *et al.* 2017). The PDBBind v2020 dataset was split into 90% training and 10% validation sets, with testing conducted on CASF-2016 and CASF-2013. For the Human datasets (Chen *et al.* 2020), we applied random splits (80% training, 10% validation, 10% test) and cold splits.

**Figure 2.**
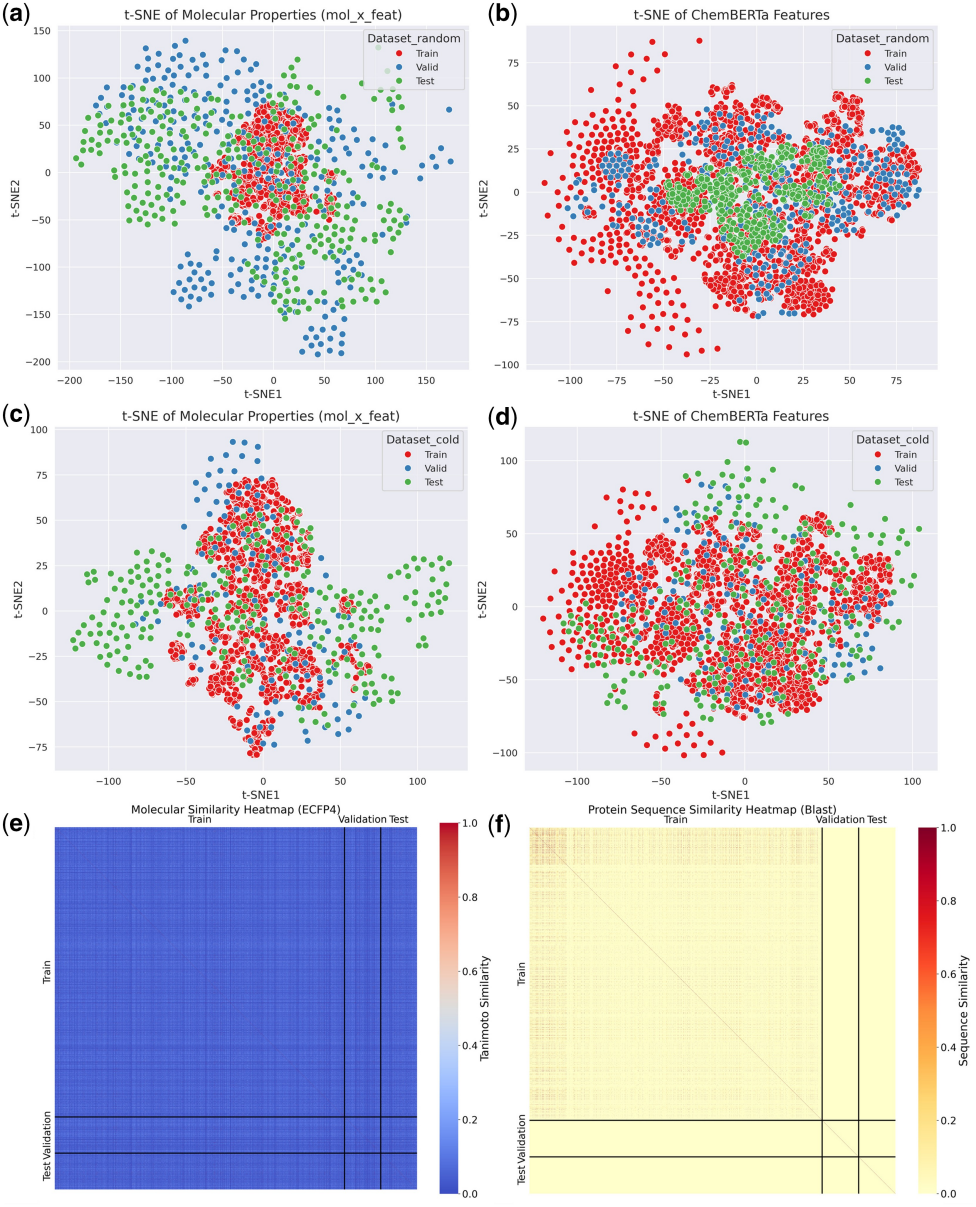
Visualization and similarity heatmap of the dataset. (a) Visualization based on physicochemical properties using t-SNE for random split of Human datasets. (b) Visualization based on ChemBERTa features using t-SNE for a random split of the Human datasets. (c) Visualization based on physicochemical properties using t-SNE for cold-pair split of Human datasets. (d) Visualization based on ChemBERTa features using t-SNE for cold-pair split of Human datasets. (e) Molecular structure similarity heatmap. (f) Protein sequence similarity heatmap.

Figure 2 and Fig. 1, available as supplementary data at *Bioinformatics* online illustrate t-SNE and PCA visualizations of molecular physicochemical properties and ChemBERTa features for random and cold splits of the Human datasets. Random splits in Fig. 2 show tightly clustered structures, while cold splits in Fig 1, available as supplementary data at *Bioinformatics* online display slight distribution separation in PCA and more pronounced separation in t-SNE, reflecting greater heterogeneity between training, validation, and test sets. Molecular similarity, assessed via ECFP4 fingerprints (Fig. 2e), is below 0.4, and protein sequence similarity, calculated using BLAST (Fig. 2f), is mostly below 0.1, confirming significant differences across sets. Figure 2, available as supplementary data at *Bioinformatics* online shows PCA with uniformly distributed features and t-SNE with local aggregation, indicating consistent data for training and testing.

## 3 Results and discussion

### 3.1 Comparative analysis of classification models

**Figure 3.**
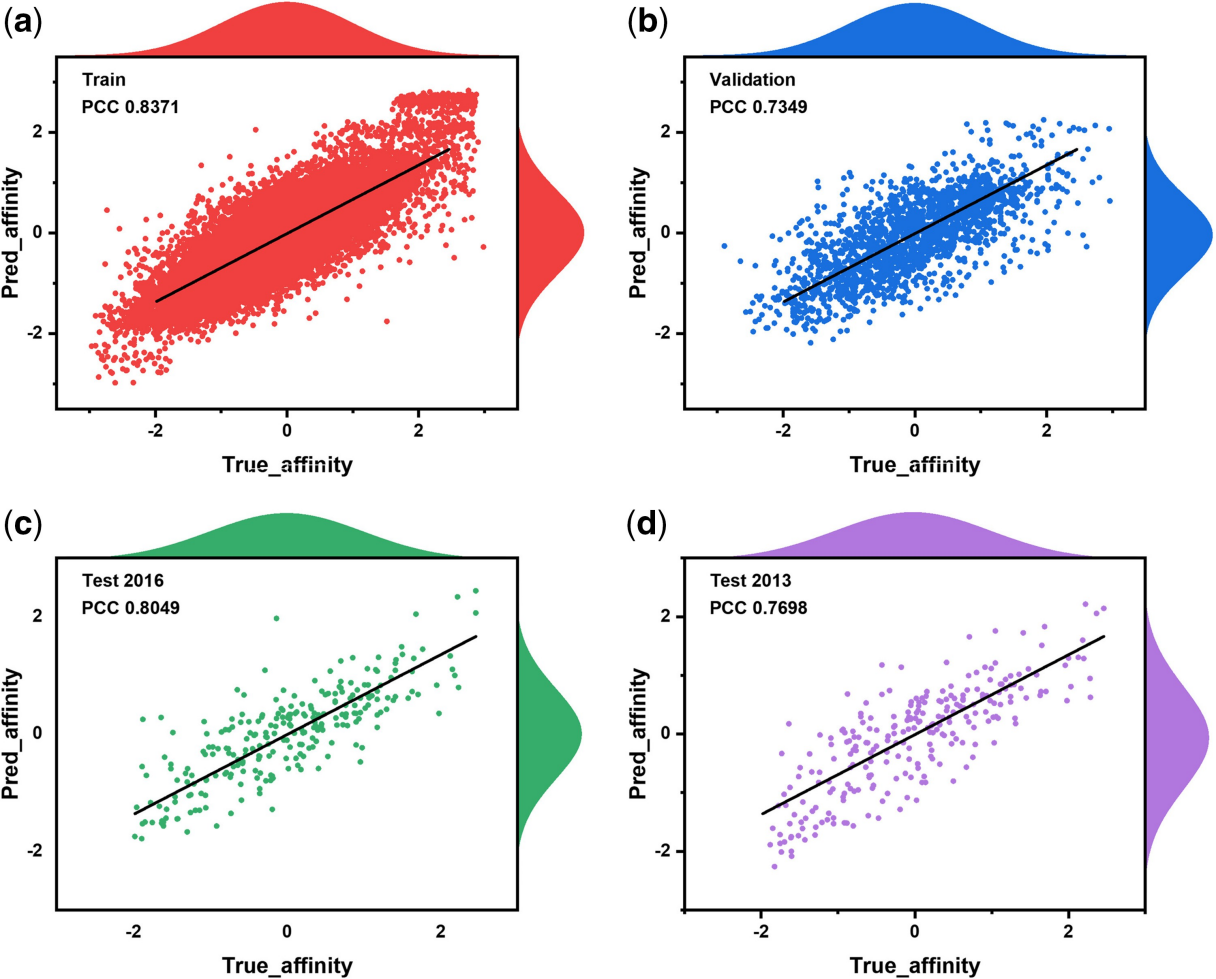
Error scatter plots for regression task. (a) Scatter plot of the error trained on PDBbind v2020. (b) Scatter plot of the error verified on PDBbind v2020. (c) Scatter plot of the error tested on CASF-2016. (d) Scatter plot of the error tested on CASF-2013.

**Figure 4.**
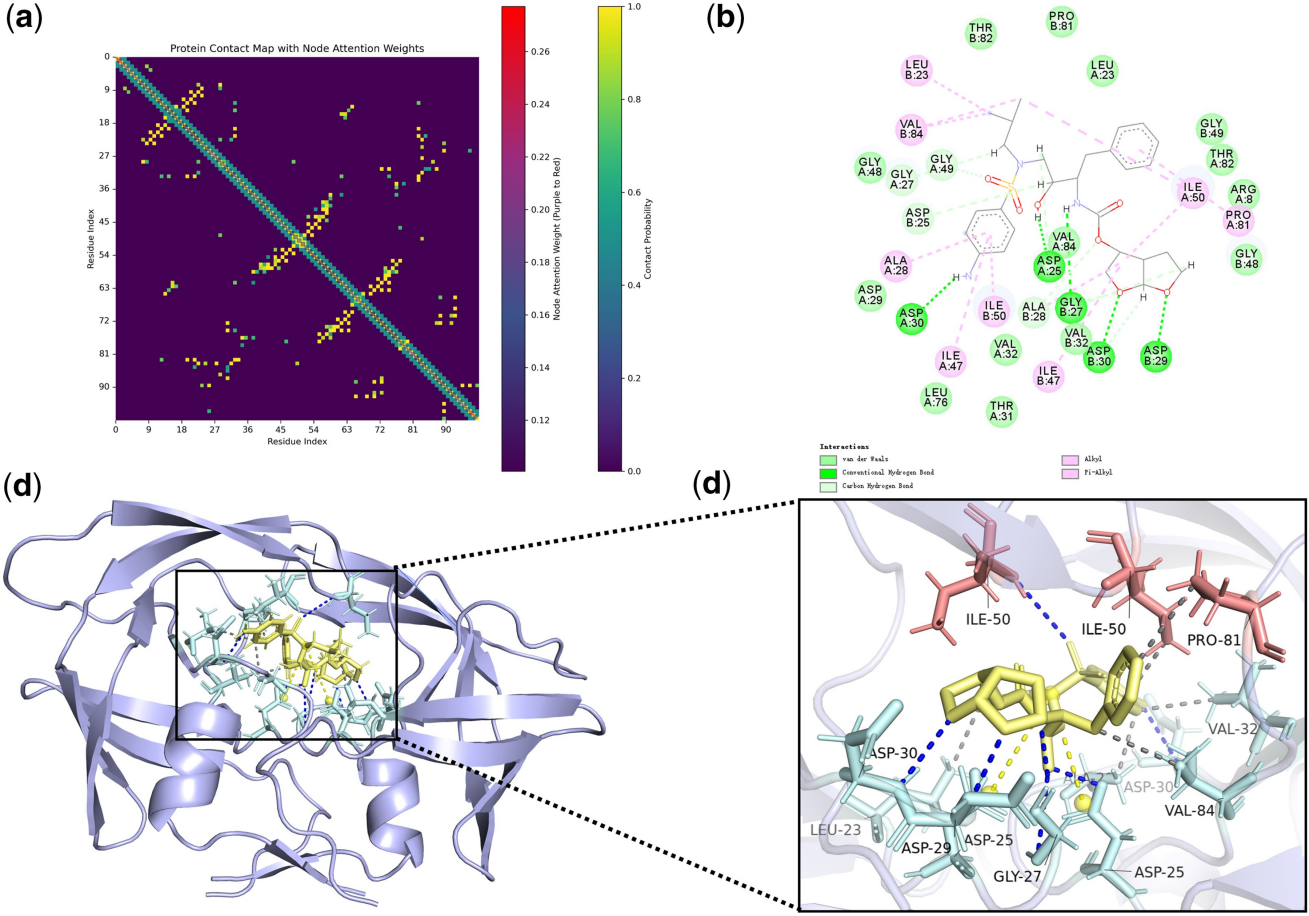
Attentional weights for molecular docking and PLXFPred recognition (3GGU). (a) Contact map of protein 3GGU, with yellow bars indicating contact strength and red bars showing attention weights of amino acid residues. (b) Interaction plane map of protein 3GGU and ligand. (c) Docking map of protein 3GGU and ligand, with pale cyan highlighting key amino acid residues interacting with the ligand. (d) Local enlarged view of protein 3GGU-ligand docking, where red indicates key amino acids identified by PLXFPred.

We evaluate PLXFPred against various deep learning and classical machine learning models, including support vector machine (SVM), random forest (RF), DeepConv-DTI (Lee *et al.* 2019), GraphDTA (Nguyen *et al.* 2021), MolTrans (Huang *et al.* 2021), DrugBAN (Bai *et al.* 2023), and DrugLAMP (Luo *et al.* 2024), using AUROC and AUPRC metrics on the Human dataset with two splitting methods, as shown in Table 1. On the randomly split training set, PLXFPred achieves an AUROC of 0.9985 and an AUPRC of 0.9986, both nearing 1 (Fig. 3, available as supplementary data at *Bioinformatics* online). The test and validation sets align closely, both exceeding 0.98, matching other deep learning models and significantly outperforming classical methods. However, hidden ligand biases in the Human dataset lead to predictions driven by pharmacological properties rather than interaction patterns, with elevated accuracy likely due to bias and overfitting rather than real-world predictive capability. To address this, we apply cold pair splitting, reducing overly optimistic performance estimates from random splitting. Under cold pair splitting, AUROC and AUPRC drop by 10%–15% across all models, with classical models like SVM and RF showing the largest decline. PLXFPred performs strongly, achieving an AUROC of 0.8608 and an AUPRC of 0.8694—both record highs (Fig. 4, available as supplementary data at *Bioinformatics* online)—outperforming the second-best model, DrugLAMP, by 0.0554 in AUPRC, a 7.6% improvement, highlighting its superior predictive capability.

**Table 1.** AUROC and AUPRC of PCXFPred and other models on random splits and cold splits on the Human dataset （Bold indicates our model.）.

Human datasets	Models	AUROC	AUPRC
**Random**	**PLXFPred**	**0.9836**	**0.9838**
	DeepConv-DTI	0.9803	0.9810
	GraphDTA	0.9808	0.9822
	MolTrans	0.9805	0.9777
	DrugBAN	0.9822	0.9802
	DrugLAMP	0.9822	0.9806
	RF	0.9518	0.9526
	SVM	0.9398	0.9204
**Cold**	**PLXFPred**	**0.8608**	**0.8694**
	DeepConv-DTI	0.8202	0.7795
	GraphDTA	0.8157	0.7704
	MolTrans	0.8038	0.76674
	DrugBAN	0.8504	0.7945
	DrugLAMP	0.8600	0.8140
	RF	0.7322	0.6701
	SVM	0.6906	0.6289

**Figure 5.**
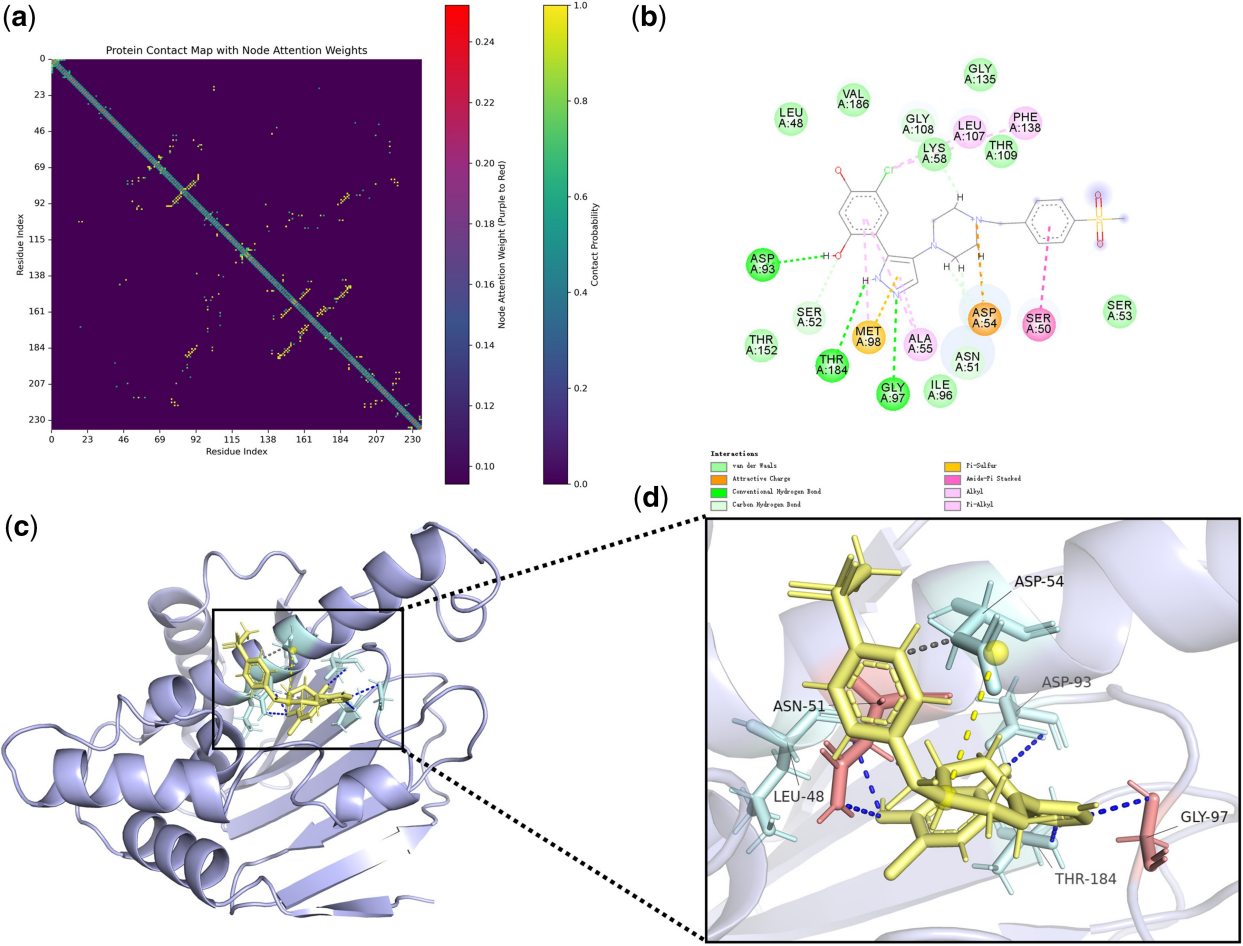
Attentional weights for molecular docking and PLXFPred recognition (2CCU). (a) Contact map of protein 2CCU, with yellow bars indicating contact strength and red bars showing attention weights of amino acid residues. (b) Interaction plane map of protein 2CCU and ligand. (c) Docking map of protein 2CCU and ligand, with pale cyan highlighting key amino acid residues interacting with the ligand. (d) Local enlarged view of protein 2CCU-ligand docking, where red indicates key amino acids identified by PLXFPred.

Additionally, the confusion matrix in Fig. 5, available as supplementary data at *Bioinformatics* online shows that PLXFPred achieves an accuracy and F1 score of 0.9315 on the randomly split test set, reflecting a strong balance between precision and recall. While slightly below the training set’s ideal values of 0.97, this demonstrates reliability and generalization in high-precision classification tasks. Cold pair splitting reduces performance, yielding accuracy and F1 scores of 0.7658 and 0.7652, respectively, due to increased data distribution complexity and class imbalance in real-world scenarios. Despite this, PLXFPred exhibits notable adaptability and robustness under cold pair splitting.

### 3.2 Comparative analysis of regression models

Figure 3 illustrates the scatter distribution of errors between actual and predicted values for the PLXFPred model across training, validation, and test sets. The scatter plot reveals that most points are light-colored and cluster near the regression line, indicating low prediction errors and strong generalization ability. In Fig. 3a, the training set yields a Pearson correlation coefficient (PCC) of 0.8371 and an R^2^ of 0.6818, reflecting a high positive correlation and good fit. The validation set shows slightly lower performance but maintains robust predictive capability, demonstrating adaptability to new data. On the CASF-2016 test set, the PCC and R^2^ are 0.8049 and 0.6224, respectively, closely aligning with training set results and confirming consistent reliability. Similarly, on the CASF-2013 test set, PLXFPred achieves a PCC of 0.7698 and an R^2^ of 0.5785, further validating its predictive strength. The distribution of absolute residuals indicates that most predictions have small residuals, with lighter hues dominating, signifying predominantly low errors. To evaluate the performance of PLXFPred in predicting protein–ligand binding affinity, we compared the model results with various state-of-the-art methods on the test sets of CASF-2016 and CASF-2013. Several of these are methods, including DeepDTA (Öztürk *et al.* 2018), DeepDTAF (Wang *et al.* 2021a,b), CAPLA (Jin *et al.* 2023), DEAttentionDTA (Chen *et al.* 2024), Pafnucy (Stepniewska-Dziubinska *et al.* 2018), GraphscoreDTA (Wang *et al.* 2023), Fusion model (Jones *et al.* 2021), IMCP-SF (Wang and Chan 2022), DLSSAffinity (Wang *et al.* 2022), PLAGCA (Shi *et al.* 2025), EM-PLA (Xie *et al.* 2025).

As shown in Table 2, PLXFPred achieves a Pearson correlation coefficient (PCC) of 0.805 on the CASF-2016 dataset, outperforming sequence-based methods such as DeepDTA (0.749), structure-based models like Pafnucy (0.722), and hybrid approaches such as the Fusion model (0.746). However, it falls slightly behind models that incorporate 3D structural information and more detailed binding site features, such as PLAGCA (0.834), EM-PLA (0.875), and DEAttentionDTA (0.845). PLXFPred also demonstrates strong ranking capability with a confidence index (CI) of 0.812, though it is marginally lower than that of models using finer information. PLXFPred’s most notable strength lies in its significant improvements across MAE, RMSE, and SD. Compared to the current top-performing models, it achieves a 44.13% increase in MAE and a 41.71% increase in RMSE over EM-PLA, while SD improves by 37.39% over PLAGCA, highlighting its superior performance in error control and prediction stability. Its R^2^ (0.6224) and adjusted R^2^ (0.6209) on CASF-2016 are closely aligned, indicating effective feature selection and minimal overfitting risk, explaining 62.24% of affinity variance without being impacted by model complexity. Overall, PLXFPred excels in predictive accuracy, error management, explanatory power, and stability.

**Table 2.** Results of PLXFPred and different models on the CASF-2016 test set (Bold indicates our model.).

Method	Pearson	CI	MAE	RMSE	SD
**PLXFPred**	**0.805**	**0.812**	**0.462**	**0.615**	**0.613**
**DeepDTA**	0.749	0.771	1.148	1.443	1.445
**DeepDTAF**	0.789	0.799	1.073	1.355	1.337
**CAPLA**	0.843	0.82	0.966	1.2	1.17
**DEAttentionDTA**	0.845	0.820	1.003	1.224	1.166
**Pafnucy**	0.722	0.776	1.253	1.553	1.434
**Fusion model**	0.746	0.773	1.207	1.513	1.454
**GraphscoreDTA**	0.831	0.819	0.981	1.249	1.216
**IMCP-SF**	0.791	0.790	1.155	1.452	1.349
**DLSSAffinity**	0.789	0.794	1.134	1.401	1.336
**PLAGCA**	0.834	0.846	0.932	1.183	0.979
**EM-PLA**	0.875	0.845	0.827	1.055	1.055

On the CASF-2013 dataset (Table 3), PLXFPred exhibits similar strengths, with improved error handling and stability, alongside reliable ranking predictions. Its R^2^ of 0.579 is slightly lower than on CASF-2016 but remains competitive. The PCC (0.77) and CI (0.787) are marginally below DEAttentionDTA’s 0.819 and 0.810, yet PLXFPred reduces MAE by 61.62%, RMSE by 52.24%, and SD by 50%, demonstrating lower error rates, greater stability, and the ability to capture diverse interaction patterns, resulting in superior overall performance.

**Table 3.** Results of PLXFPred and different models on the CASF-2013 test set (Bold indicates our model.).

Method	Pearson	CI	MAE	RMSE	SD
**PLXFPred**	**0.770**	**0.787**	**0.423**	**0.649**	**0.646**
**DeepDTA**	0.654	0.731	1.417	1.785	1.790
**DeepDTAF**	0.672	0.732	1.151	1.791	1.780
**CAPLA**	0.770	0.780	1.154	1.446	1.436
**DEAttentionDTA**	0.819	0.810	1.102	1.359	1.292
**Pafnucy**	0.687	0.748	1.355	1.666	2.242
**Fusion model**	0.741	0.777	1.554	1.252	1.523
**GraphscoreDTA**	0.758	0.780	1.235	1.545	1.479
**DLSSAffinity**	0.732	0.746	1.304	1.596	1.584

### 3.3 Interpretability of attention visualization

PLXFPred uses graph attention convolutional layers, graph attention pooling layers, and cross-attention mechanisms to generate multi-head attention weights for ligands and target residues, enabling the calculation of weights assigned to target residues and ligand atoms. These weights reveal their relative importance in the prediction process. To demonstrate, we analyzed two representative protein–ligand pairs. The first involves darunavir, an HIV protease inhibitor (3GGU), with a predicted affinity of 9.77 by PLXFPred (Sasková *et al.* 2009). The second is an Hsp90 inhibitor (2CCU), a human pyrazole compound with piperazine, morpholino, and piperidinyl structures, exhibiting a predicted affinity of 6.13 (Barril *et al.* 2006).

**Figure 6.**
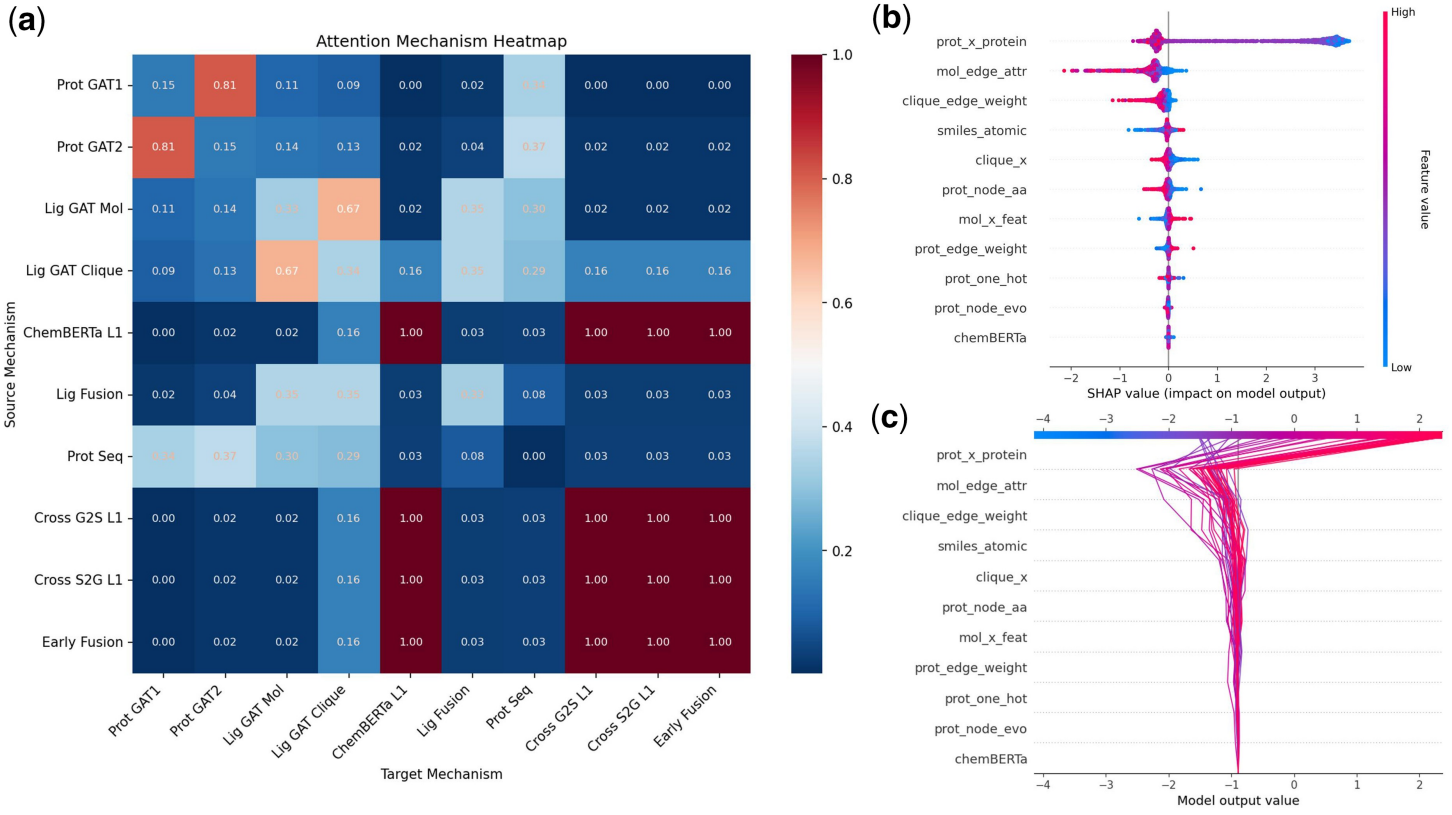
Attention and feature analysis. (a) Attention heat map (Prot GAT1: Protein Graph Attention Network Layer 1. Prot GAT2: Protein Graph Attention Network Layer 2. Lig GAT Mol: Ligand Molecular Graph Attention Network. Lig GAT Clique: Ligand Clique Graph Attention Network. ChemBERTa L1: ChemBERTa Model Layer 1 Output. Lig Fusion: Ligand Fusion Features. Prot Seq: Protein Sequence Features. Cross G2S L1: Graph-to-Sequence Cross Transformer Layer 1. Cross S2G L1: Sequence-to-Graph Cross Transformer Layer 1. Early Fusion: Early Fusion Features). (b) Density scatter plot of SHAP feature analysis. (c) Decision diagram of SHAP feature analysis.

The interaction between protein 3GGU and its ligand, along with the attention weights from PLXFPred, is illustrated in Fig. 4. Figure 4a and Fig. 6a, available as supplementary data at *Bioinformatics* online display the attention weights of amino acid residues and edges, where a deeper red hue indicates higher attention weights for amino acids, and a more intense yellow hue reflects stronger contact strength. High attention weights are visualized in the docking of protein 3GGU with the ligand in Fig. 4c and d and the 3GGU pocket-ligand docking in Fig. 6b, available as supplementary data at *Bioinformatics* online. In Fig. 4c, pale cyan highlights key amino acid residues in 3GGU, while Fig. 4d and Fig. 6b, available as supplementary data at *Bioinformatics* online mark residues with higher weights identified by PLXFPred in red. Notably, the ILE-50 residue on the α chain (Fig. 4d) forms a hydrogen bond with the oxygen of the S = O double bond in the ligand’s sulfate group, with weights of 2 for the S = O bond and 0.34 for the oxygen atom, as shown in Fig. 6c and d, available as supplementary data at *Bioinformatics* online. The ILE-50 residue on the β chain engages in a hydrophobic interaction with the ligand’s benzene ring, with weights of 0.35 for the atoms and 1.5 for the bonds. Additionally, Fig. 4b reveals a hydrophobic interaction with the terminal -CH3 group, which has the highest weight of 0.49 among all atoms. The PRO-81 residue also forms a hydrophobic interaction with the benzene ring. Furthermore, PLXFPred identifies critical interactions in the protein pocket, such as ASP 28-GLY 27 and ASP 29-ASP 30, where residues interact with oxygen atoms on the bifuran ring, with weights of 0.37 and 0.40, and an NH-H interaction with a weight of 0.43. Residue pairs like ASN 83-PRO 81 and ASN 83-THR 82 contribute weak forces, supporting pocket formation and stability.

The interaction between protein 2CCU and its ligand, along with PLXFPred’s attention weights, is depicted in Fig. 5. The local docking diagram (Fig. 5d) highlights GLY 97 and ASN 51 as the most significant amino acid residues. The nitrogen atom on the ligand’s pyrrolidine ring forms a hydrogen bond with GLY 97, with a weight of 0.36 for the nitrogen and 1.5 for the connected edge (Fig. 7d and c, available as supplementary data at *Bioinformatics* online). ASN 51, together with LEU 48, forms a hydrogen bond with the -OH group on the benzene ring, with a weight of 0.47, second only to the -Cl weight of 0.5 on the ligand’s benzene ring, and forms a hydrophobic force with it. The SER 50-ASN 51 edge, where the amide group of SER 50’s main chain participates in amide-pi stacking with the ligand’s aromatic ring, is a key interaction identified by PLXFPred (Fig. 5 and Fig. 7b, available as supplementary data at *Bioinformatics* online). Though a weak non-covalent force, this stacking enhances binding selectivity and stability. ASN 51 also forms a weak hydrogen bond with the benzene ring, while the interaction with ASP 54 boosts overall binding affinity through a synergistic effect. The aforementioned research demonstrates that the PLXFPred model can infer important atoms and chemical bonds in ligands and important residues in proteins through attention weights.

### 3.4 Ablation experiment

Ablation studies on PLXFPred’s nine core modules confirm their contributions to model performance (Zhai *et al.* 2025). Combined with Table 4, the ProteinGraphEncoder is critical, with its removal causing an 18.6% PCC drop, 54.05% and 23.62% increases in RMSE, and a 7.6% F1 score reduction, highlighting its role in capturing protein structural features via graph neural networks. The WeightedMSELoss module is also vital, optimizing key samples and preventing overfitting; its absence leads to a 13.04% PCC drop, 37.84% MSE increase, and 17.26% RMSE rise. The CrossTransformerFusion module, though less impactful, supports feature integration, with its removal reducing PCC by 1.74% and increasing MSE and RMSE by 5.41% and 1.79%. Early Fusion modestly affects regression but notably impacts classification, lowering ROC-AUC and F1 by 6.0% and 3.0%, aiding in identifying key interactions like hydrogen bonding. Data processing enhances robustness, with its removal decreasing PCC by 0.62% and increasing MSE and RMSE by 8.11% and 3.26%.

**Table 4.** The impact of ablation of key modules in PLXFPred on the model (Bold indicates our model.).

Method	PCC	CI	MAE	MSE	RMSE	Accuracy	ROC-AUC	F1	AU PRC
**Baseline**	**0.805**	**0.812**	**0.462**	**0.378**	**0.615**	**0.9315**	**0.9829**	**0.9316**	**0.9835**
**ProteinGraph Encoder**	0.655	0.735	0.582	0.576	0.759	0.8607	0.95	0.8605	0.9487
**LigandGraph Encoder**	0.749	0.780	0.532	0.469	0.685	0.9005	0.9588	0.9004	0.9558
**ProteinSequenceEncoder**	0.798	0.801	0.479	0.368	0.606	0.9055	0.9709	0.9054	0.9728
**LigandSequenceEncoder**	0.762	0.780	0.532	0.469	0.685	0.9005	0.9588	0.9004	0.9558
**Early_fusion**	0.791	0.797	0.488	0.386	0.621	0.8756	0.9535	0.8756	0.9607
**CrossTransformerFusion**	0.791	0.796	0.485	0.391	0.625	0.9080	0.9659	0.9080	0.9623
**Data_processing**	0.80	0.805	0.479	0.402	0.634	0.9008	0.9663	0.9007	0.9651
**Multitask_weighting**	0.757	0.794	0.542	0.496	0.705	0.8980	0.9723	0.8978	0.9723
**Weighted__MSELoss**	0.700	0.756	0.567	0.519	0.720	0.9025	0.9710	0.9024	0.9687

In summary, the PLXFPred excels in predicting protein–ligand interactions and affinities through its multi-modal feature fusion and multi-task learning framework. The ProteinGraphEncoder and LigandGraphEncoder capture complex structural features, while the CrossTransformerFusion module enhances predictive accuracy by integrating diverse data sources. Early fusion boosts classification by improving subtle category distinctions, and weighted loss functions address imbalanced data, collectively driving PLXFPred’s superior performance.

### 3.5 Shap

The attention heatmap (Fig. 6a) visualizes feature interactions across target (horizontal) and source (vertical) mechanisms. Notably, Prot_GAT1 assigns a high attention weight of 0.81 to key protein residues, indicating its focus on localized binding sites critical for affinity prediction. In contrast, Prot_GAT2 exhibits a more distributed attention pattern (weights ranging from 0.37 to 0.14), capturing long-range interactions and allosteric regulatory networks that contribute to global structural stability. This dual focus enhances the model’s ability to integrate direct binding features with broader conformational dynamics, as evidenced by the complementary attention distributions. For the ligand side, lig_GAT_mol and lig_GAT_Clique share a strong interaction weight of 0.67, reflecting the importance of chemical properties and substructural motifs in ligand encoding. The elevated attention weights of Early_fusion, Cross_S2G_L1, Cross_G2S_L1, and ChemBERTa_L1 (up to 1.0) reflect a multi-layered Cross_S2G_L1 and Cross_G2S_L1 dynamically refine this scaffold through sequence-graph interplay, and ChemBERTa_L1 enriches it with domain-specific knowledge. This collective high attention underscores their collaborative role in driving the model’s predictive accuracy and interpretability.

The SHAP scatter plot (Fig. 6b) uses a bee-swarm visualization; Prot_x_protein stands out with a wide SHAP range, reflecting its significant and variable role in capturing global protein sequence patterns. Despite a modest impact on ablation, its integration via the CrossTransformer, with attention weights of 0.29–0.37 to Prot_GAT1, Prot_GAT2, Lig_GAT_Mol, and Lig_GAT_Clique, enhances protein–ligand interaction modeling. Ligand features like mol_edge_attr and smiles_atomic show narrower SHAP ranges, indicating a secondary role, likely due to the dominant influence of protein structural complexity on interaction dynamics. The decision diagram (Fig. 6c) ranks features by mean absolute SHAP. prot_x_protein reaffirms its primacy with the highest mean SHAP, encoding critical global and sequential information that sets the predictive baseline. Mol_edge_attr follows as a key secondary predictor, emphasizing ligand connectivity’s role in steric and electrostatic constraints. Auxiliary features like clique_edge_weight and smiles_atomic contribute to adding physical and chemical constraints. The diagram’s layered structure reveals hierarchical dependencies. For instance, high SHAP bars for prot_x_protein often correlate with downstream adjustments from ligand features like clique_edge_weight and smiles_atomic, which impose physical and chemical boundaries essential for accuracy. Quantitatively, these auxiliary features account for approximately 28.3% of total SHAP variance, ensuring the model avoids overgeneralization in diverse chemical spaces.

In conclusion, the detailed examination of the attention mechanism heat map, SHAP analysis, and decision diagram reveals that the PLXFPred model exhibits a hierarchical multi-level feature interaction mechanism in predicting protein–ligand affinity. Protein sequences form the core of the global feature prediction framework, with key amino acids identified via graph attention, while ligand structural characteristics enable precise local adjustments. This “global dominance-local correction” model aligns closely with the characteristics of biomolecular interactions.

## 4 Conclusions

In this work, we introduce PLXFPred, a deep learning framework for protein–ligand interaction prediction, integrates BILSTM, GAT, and advanced fusion mechanisms to achieve over 50% error reduction (MSE, RMSE, SD) compared to traditional models, while remaining competitive with advanced deep learning approaches. Its interpretable design, leveraging attention weights, heat maps, and SHAP analysis, reveals hierarchical feature interactions, with proteins as primary drivers and ligands as auxiliary factors, aligning with biomolecular patterns. With its innovative multi-modal fusion architecture and interpretable design, PLXFPred achieves breakthroughs in both performance and interpretability for protein–ligand interaction prediction. This framework not only offers a more accurate computational tool for structural biology but also provides theoretical insights into molecular recognition mechanisms, supporting target identification and rational ligand optimization.

## Supplementary Material

btaf662_Supplementary_Data
