# Supplementary material for: PLXFPred: interpretable cross-attention networks with hierarchical fusion of multi-modal features for predicting protein–ligand interactions and affinities
Source: Bioinformatics. 2026 Jan 9;42(1):btaf662. doi: 10.1093/bioinformatics/btaf662 (PMC12936868; doi:10.1093/bioinformatics/btaf662)
Supplement: btaf662_Supplementary_Data [file btaf662_supplementary_data.zip › Supplementary Materials.docx]

**Supplementary Materials**

**Title:** PLXFPred: Interpretable cross-attention networks with hierarchical fusion of multimodal features for predicting protein-ligand interactions and affinities.

**Feature extraction**

The process of feature extraction for proteins involves converting each amino acid in the sequence into a one-hot encoding and identifying its classification features, like whether it is aliphatic or aromatic, as well as its physical and chemical properties, such as molecular weight, isoelectric point, and hydrophilicity. The multidimensional scaling method proposed by Venkatarajan and Braun was also used, with the specific parameters shown in Table S1 [1]. This method simplified the physicochemical properties of 237 proteins into 5 amino acid quantitative descriptors. The ESM-2 pre-trained model was used to obtain 1280 features from protein sequences and predict the probability of amino acids being close to each other to generate a contact map, which was used for the construction and training of the graph neural network [2].

The feature extraction of ligands uses RDKit to generate molecular objects from standardized SMILES strings [3]. The nodes of the molecular graph are atoms, and their features include the degree of the atom, the number of implicit hydrogen atoms of the atom, the hybridization type of the atom, whether the atom is an aromatic atom, and the stereochemical information of the atom. One-hot encoding is used to map the chemical bond type between each pair of atoms into a number as an edge feature. The molecular graph is decomposed into clusters via a tree to extract higher-level structural information. In addition, the pre-trained model ChemBERTa-zinc-base-v150 is used to extract the 768-dimensional global features of the ligand [4].All the features finally extracted are shown in Table S2.

**Clustering-based pair split strategy**

Against the cold pair segmentation of the HUMAN dataset, we use a binarized ECFP4 feature to represent ligand, and an integral PSC feature to characterize proteins. To accurately measure the pairwise distance, we use the Jaccard distance for ECFP4 and the cosine distance for PSC, respectively. We choose γ=0.5 in both ligand and protein clusterings since this choice can prevent over-large clusters and ensure separate dissimilar samples.where 5% and 10% of drug-target interaction (DTI) pairs were assigned to validation and test sets, respectively, and removed all drugs and proteins associated with them were removed from the training set. Cold splitting ensures test drugs and proteins are unseen during training, preventing reliance on known features and enhancing model robustness.

**Data preprocessing**

There are two main parts in data processing. One part is data preprocessing, which mainly uses the Z-score statistical method to remove outliers in the regression task during the data cleaning stage. To fix the issue of unevenly distributed classified data, the SMOTE oversampling method balances the categories, which helps reduce the model's bias [5]. Additionally, Min-Max normalization standardizes the affinity data in the regression task to guarantee the consistency of the feature value range. The other part is data enhancement, which includes adding noise, random masking, random rearrangement of fragments, and random rotation and reversal of images to increase the data diversity and improve the model's generalization ability. Additionally, we set up a DynamicEdgeConv module to dynamically learn the feature representation of the graph's edges, as illustrated in Formula S1. By changing the features or weights of the edges based on the node features, the graph's structural information can be captured more easily, which enhances the understanding and handling of graph data.

$e_{ij}=\mathrm{Sigmoid}(W_{2}\cdot ReLU(W_{1}\left[ x_{i} | \left| x_{j} \right]+b_{1} \right)+b_{2})$ (1)

**Data availability**

The experimental data used in this work are available at <https://github.com/xiyuyangtuo/PLXFPred/>. All data are from public resources. The data source of Human can be obtained at https://github.com/lifanchen-simm/transformerCPI/blob/master/Human%2CC.elegans/dataset/human_data.txt, and PDBbind-v2020, CASF-2013, and CASF-2016 can be obtained at https://www.pdbbind-plus.org.cn/. The pre-trained models esm2_t33_650M_UR50D.pt and ChemBERTa-zinc-base-v1 used were obtained from https://github.com/facebookresearch/esm and https://huggingface.co/seyonec/ChemBERTa-zinc-base-v1, respectively. The co-crystalized ligands from PDB are available at https://www.rcsb.org by searching their PDB IDs.


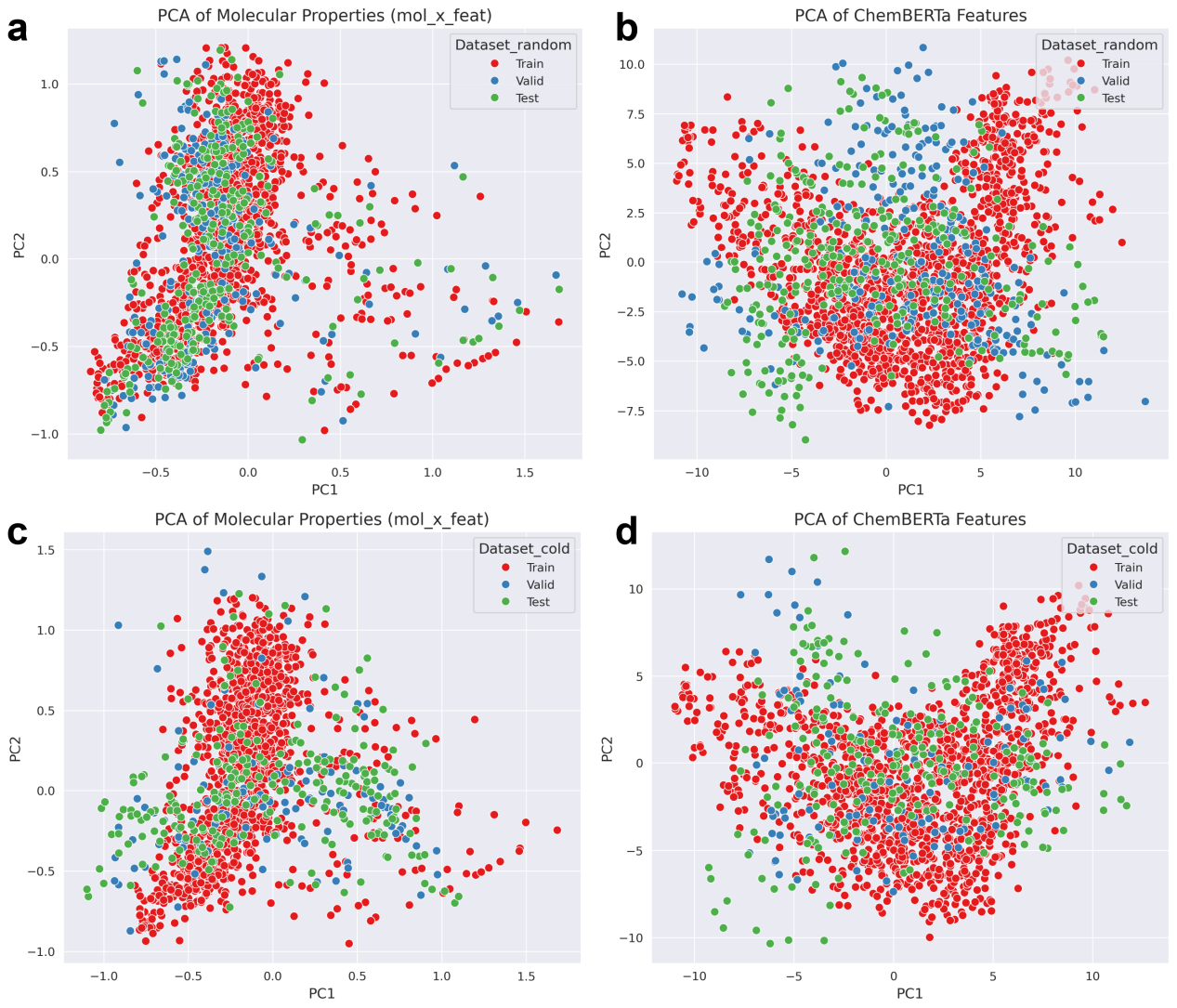


**Figure S1** Visualization of datasets (PCA). (a) Visualization of human dataset based on physicochemical properties using PCA at random split. (b) Visualization of human dataset based on ChemBERTa features using PCA at random split. (c) Visualization of human dataset based on physicochemical properties using PCA at cold pair split. (d) Visualization of human dataset based on ChemBERTa features using PCA at cold pair split.


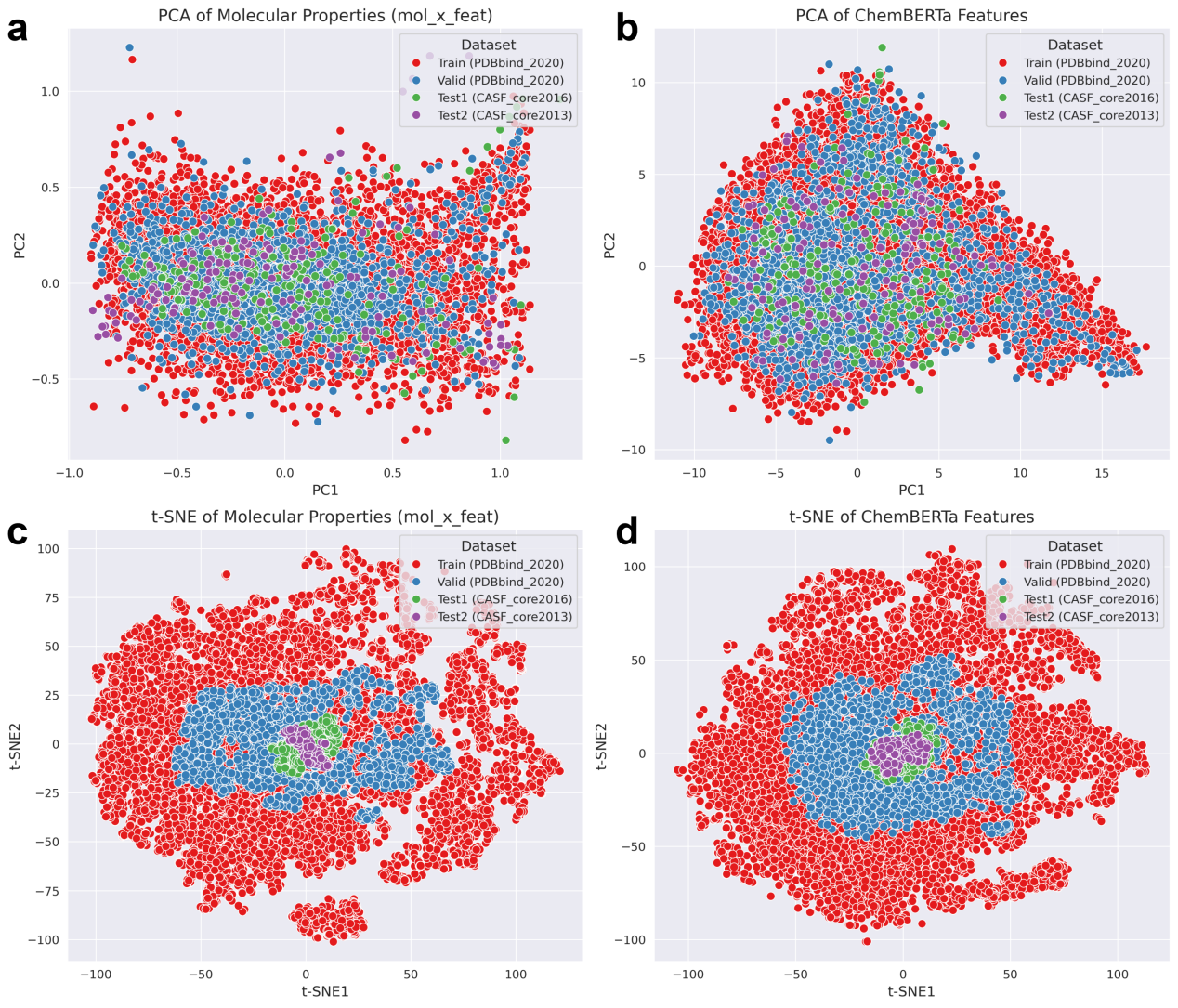


**Figure S2** Visualization of datasets (PDBbind). (a) Visualization of physicochemical properties using PCA under random split of PDBbind dataset. (b) Visualization of ChemBERTa properties using PCA under random split of PDBbind dataset. (c) Visualization of physicochemical properties using t-SNE under random split of PDBbind dataset. (d) Visualization of ChemBERTa properties using t-SNE under random split of PDBbind dataset.


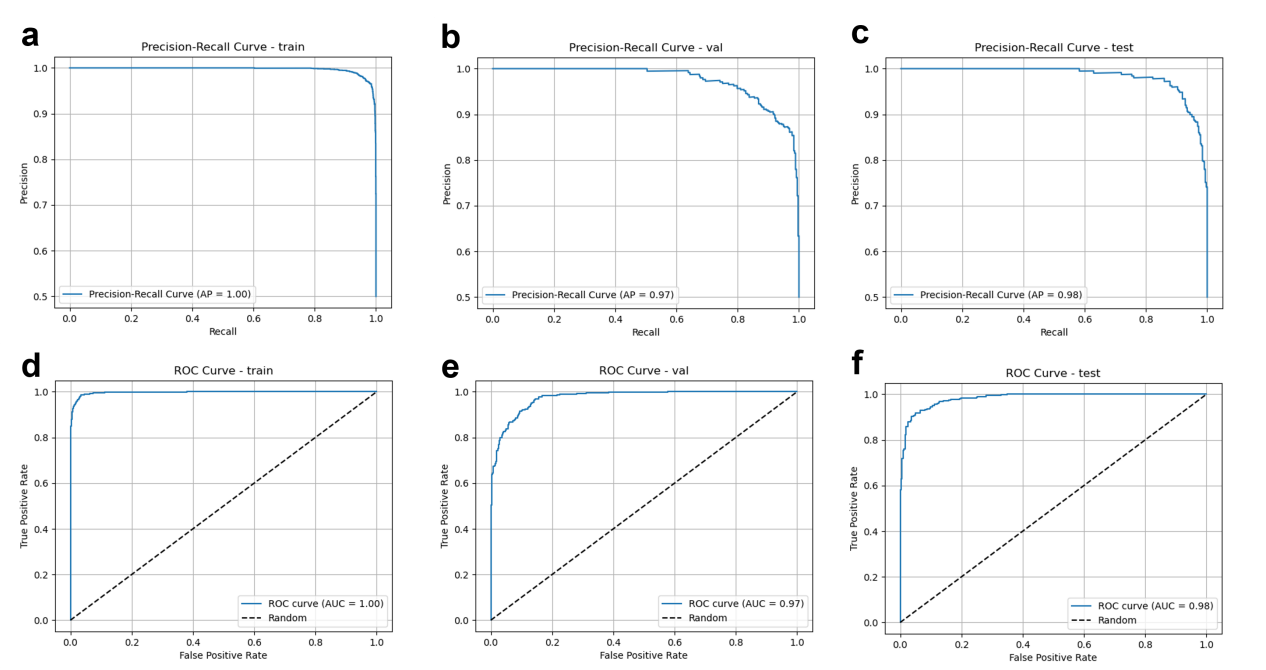


**Figure S3** AUPRC and AUROC of the model PLXFPred on a randomly assigned human dataset. (a)(d) AUPRC and AUROC on the training set. (b)(e) AUPRC and AUROC on the validation set. (c)(f), AUPRC and AUROC on the test set.


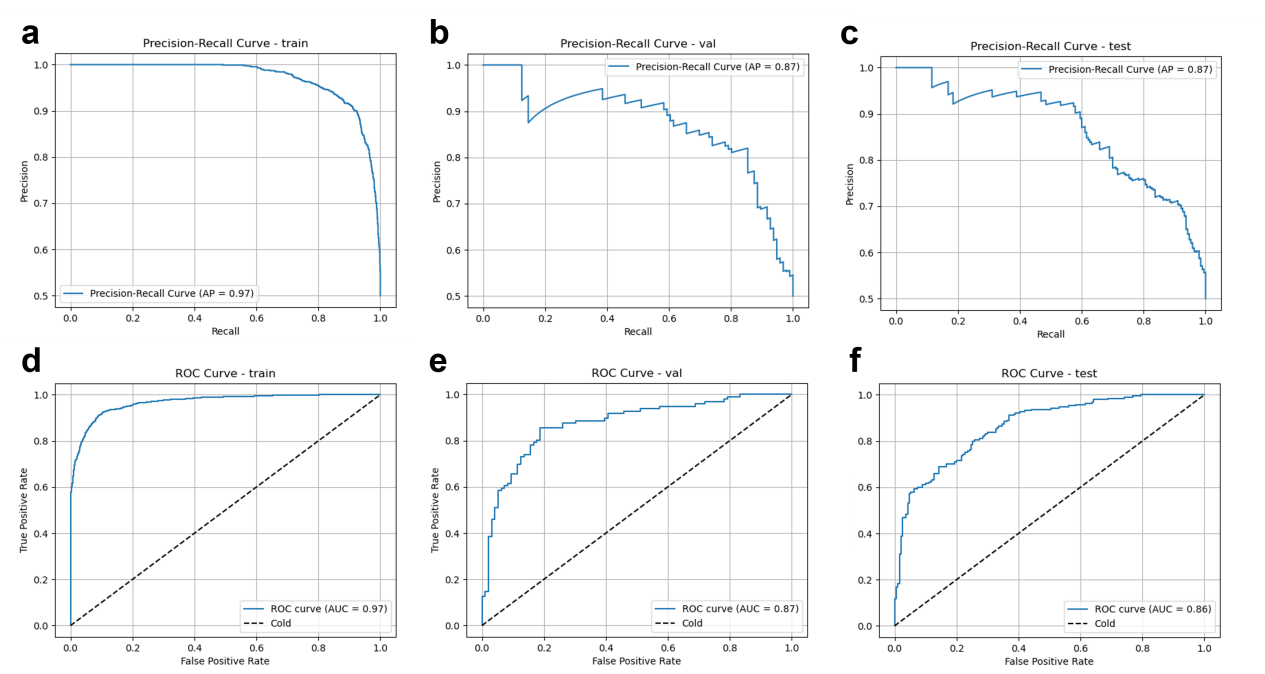


**Figure S4** AUPRC and AUROC of the model PLXFPred on the cold-pair assigned human dataset. (a)(d) AUPRC and AUROC on the training set. (b)(e) AUPRC and AUROC on the validation set. (c)(f) AUPRC and AUROC on the test set.


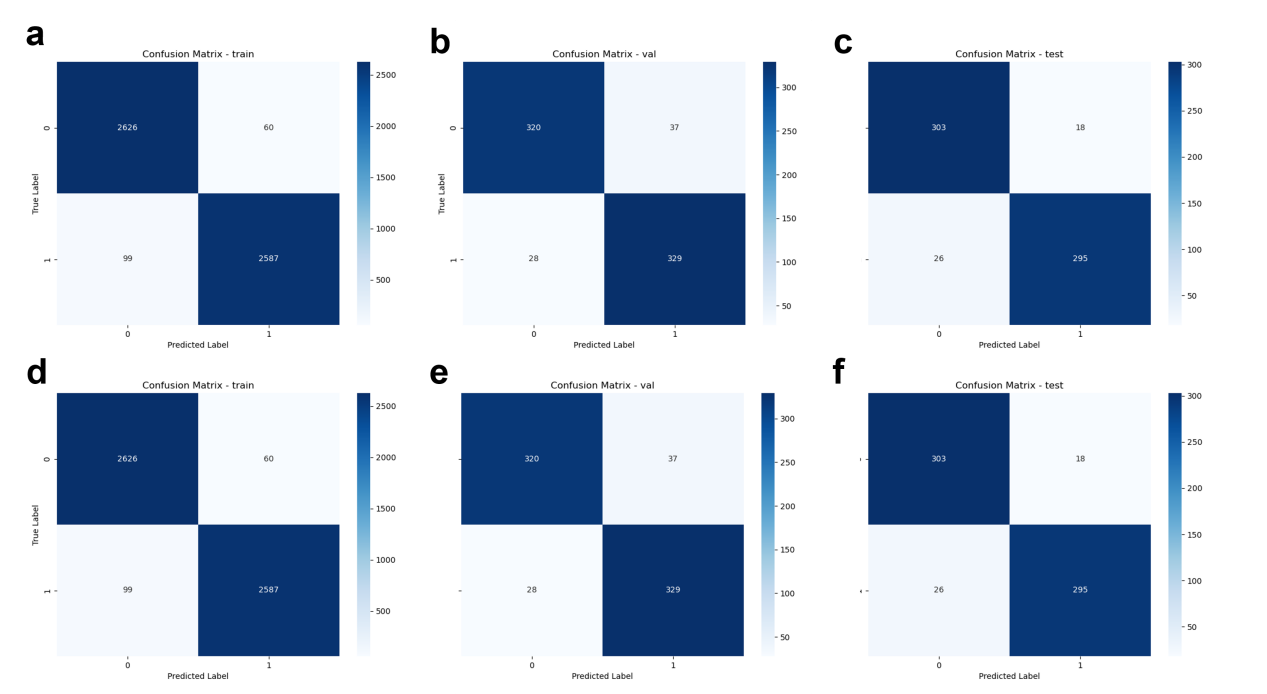


**Figure S5** Confusion Matrix. (a)(b)(c) They are respectively the training set, validation set and test set of the randomly split human dataset. (d)(e)(f) They are the training set, validation set and test set on the cold segmentation human dataset.


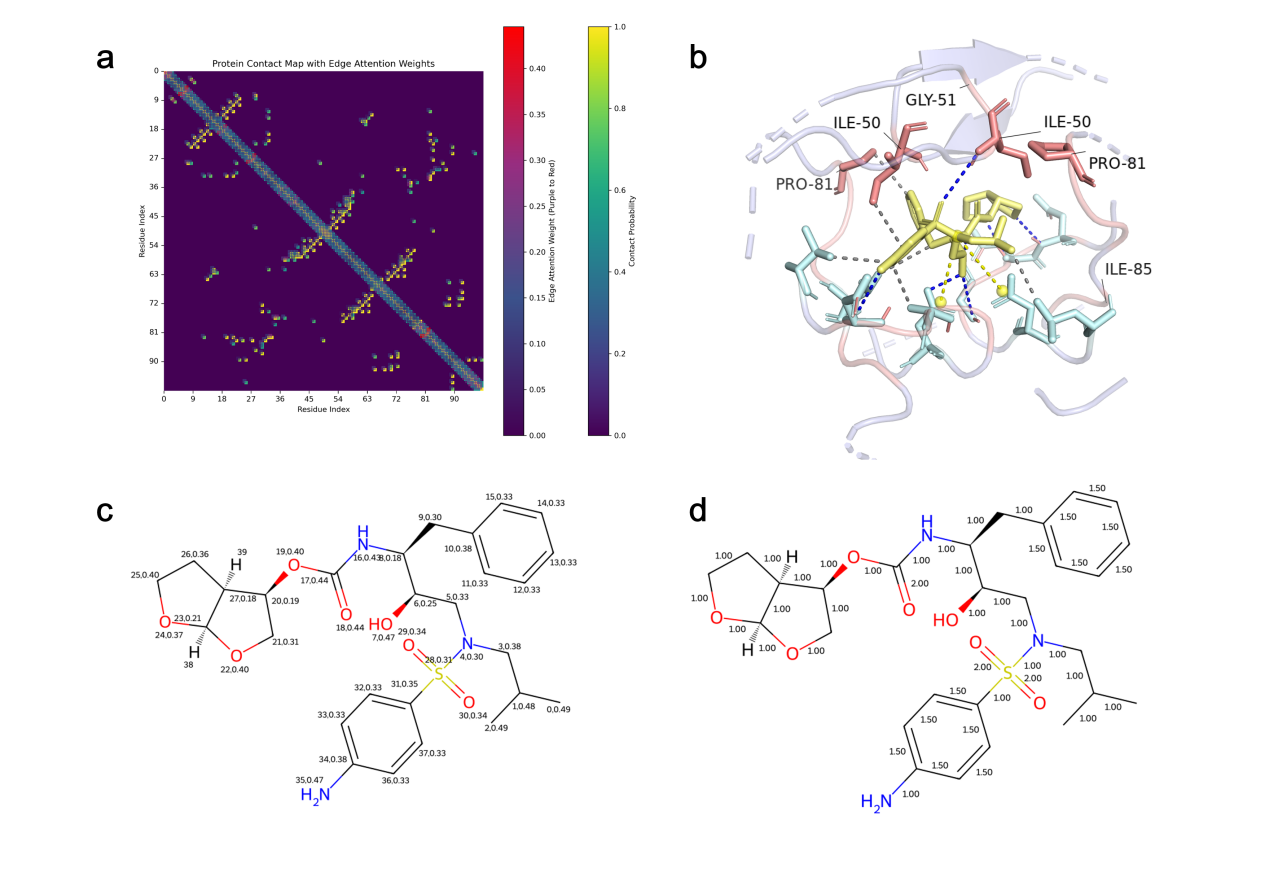


**Figure S6** Display of attention weights of proteins and ligands (3GGU). (a) 3GGU protein contact map, the yellow bar is the contact strength, and the red bar is the attention weight of the amino acid edge. (b) 3GGU protein pocket and ligand interaction map, the key amino acids and edges identified by PLXFPred are red, blue is hydrogen bonds, gray is hydrophobic forces, and yellow is salt bridges. (c)(d) PLXFPred identifies the weights of each point and edge of the ligand.**
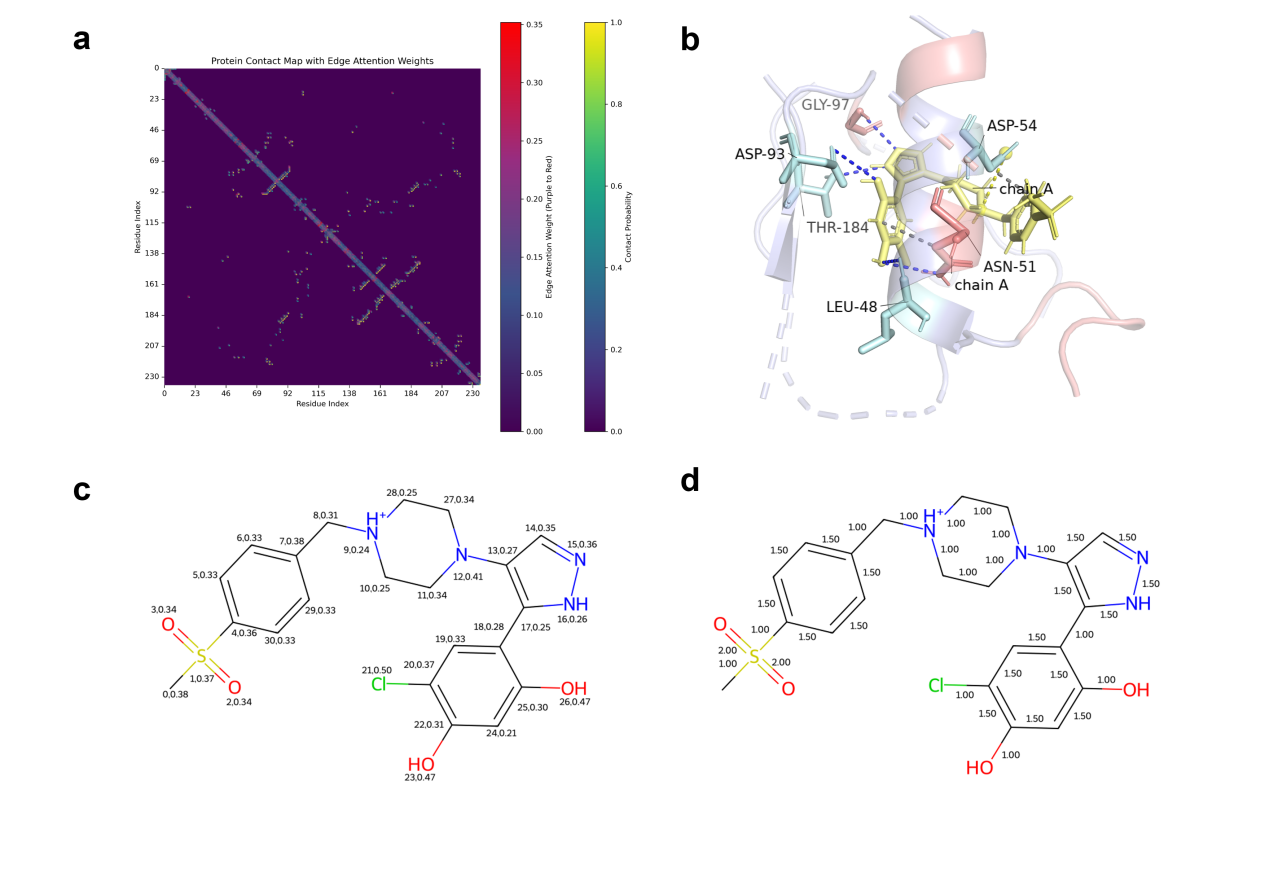
**

**Figure S7** Display of attention weights of proteins and ligands (2CCU). (a) 2CCU protein contact map, the yellow bar is the contact strength, and the red bar is the attention weight of the amino acid edge. (b) 2CCU protein pocket and ligand interaction map, the key amino acids and edges identified by PLXFPred are red, blue is hydrogen bonds, gray is hydrophobic forces, and yellow is salt bridges. (c)(d) PLXFPred identifies the weights of each point and edge of the ligand.

**Table S1** Physicochemical Properties of 20 Amino Acids.

| **Amino Acids** | **E1** | **E2** | **E3** | **E4** | **E5** |
| --- | --- | --- | --- | --- | --- |
| **A** | 0.008 | 0.134 | -0.475 | -0.039 | 0.181 |
| **R** | 0.171 | -0.361 | 0.107 | -0.258 | -0.364 |
| **N** | 0.255 | 0.038 | 0.117 | 0.118 | -0.055 |
| **D** | 0.303 | -0.057 | -0.014 | 0.225 | 0.156 |
| **C** | -0.132 | 0.174 | 0.070 | 0.565 | -0.374 |
| **Q** | 0.149 | -0.184 | -0.030 | 0.035 | -0.112 |
| **W** | 0.221 | -0.280 | -0.315 | 0.157 | 0.303 |
| **G** | 0.218 | 0.562 | -0.024 | 0.018 | 0.106 |
| **H** | 0.023 | -0.177 | 0.041 | 0.280 | -0.021 |
| **I** | -0.353 | 0.071 | -0.088 | -0.195 | -0.107 |

**Table S2** All features extracted by PLXFPred.

|  | **Feature Name** | **Feature Description** |
| --- | --- | --- |
| **Protein** | Prot_node_aa | Physicochemical characteristics of proteins |
|  | Prot_one_hot | One-hot encoding of each amino acid |
|  | Prot_edge_weight | Protein edge weights |
|  | Prot_node_env | Features extracted by the ESM2 model |
|  | Prot_x_protein | Multidimensional annotation of amino acids |
| **Ligand** | Mol_x_feat | Physical and chemical characteristics of atoms |
|  | Mol_edge_attr | Features of atomic edges |
|  | Clique_x | Characteristics of the group |
|  | Clique_edge_attr | Weight of cluster edges |
|  | Smiles_atomic | Standardization of smiles |
|  | ChemBERTa | Features extracted by the ChemBERTa model |

**References**

1. Venkatarajan MS, Braun W. New quantitative descriptors of amino acids based on multidimensional scaling of a large number of physical-chemical properties, Journal of Molecular Modeling 2001;7:445-453.

2. Rives A, Meier J, Sercu T et al. Biological structure and function emerge from scaling unsupervised learning to 250 million protein sequences, Proceedings of the National Academy of Sciences of the United States of America 2021;118.

3. Bento AP, Hersey A, Félix E et al. An open source chemical structure curation pipeline using RDKit, Journal of Cheminformatics 2020;12.

4. Chithrananda S, Gr G, Ramsundar B. ChemBERTa: Large-Scale Self-Supervised Pretraining for Molecular Property Prediction, Arxiv 2020.

5. Bao Y, Yang SB. Two Novel SMOTE Methods for Solving Imbalanced Classification Problems, IEEE ACCESS 2023;11:5816-5823.
