# Supplementary figures and images for: PLXFPred: interpretable cross-attention networks with hierarchical fusion of multi-modal features for predicting protein–ligand interactions and affinities
Source: Bioinformatics. 2026 Jan 9;42(1):btaf662. doi: 10.1093/bioinformatics/btaf662 (PMC12936868; doi:10.1093/bioinformatics/btaf662)

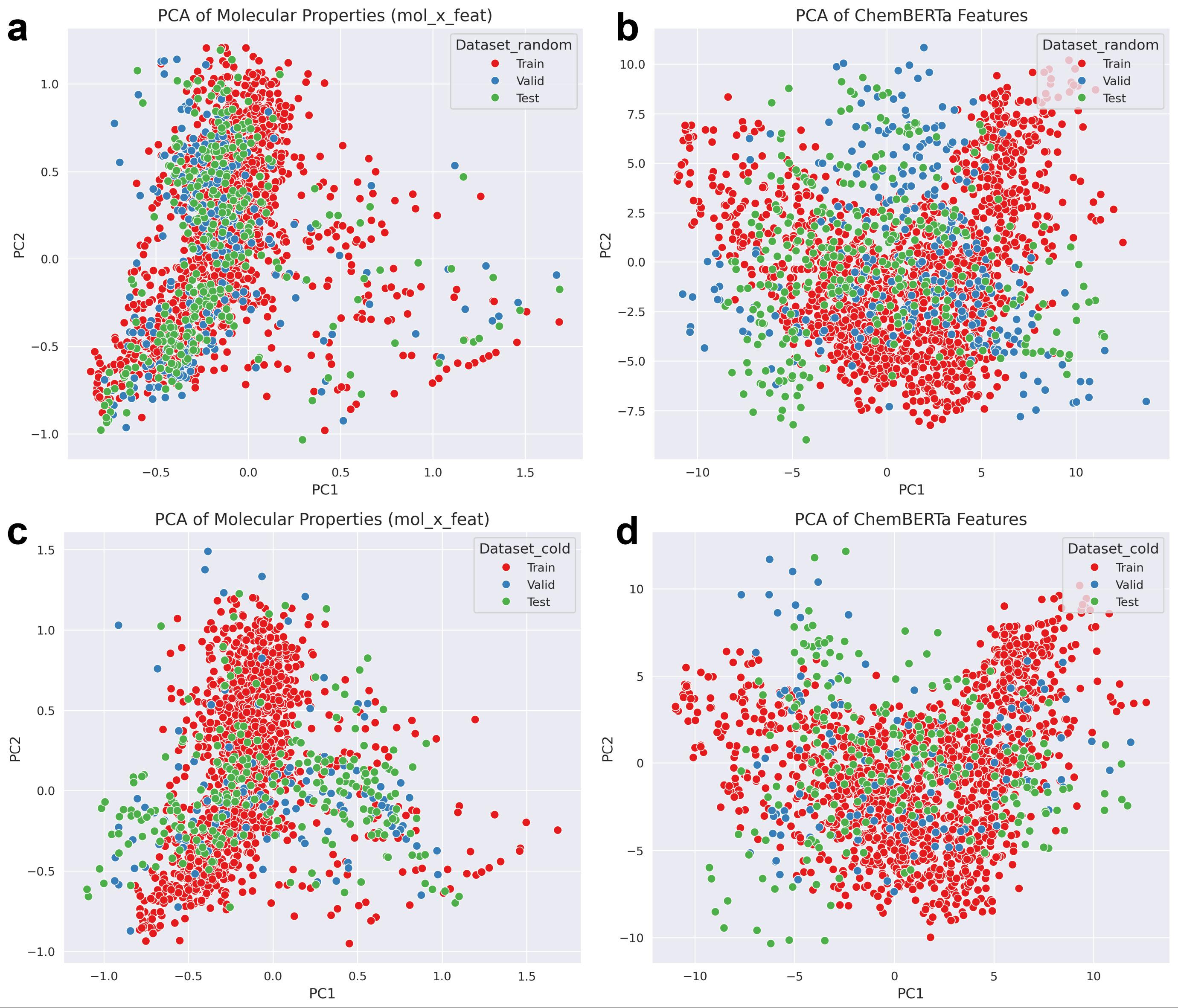

Supplement: btaf662_Supplementary_Data [file btaf662_supplementary_data.zip › figS1.png]

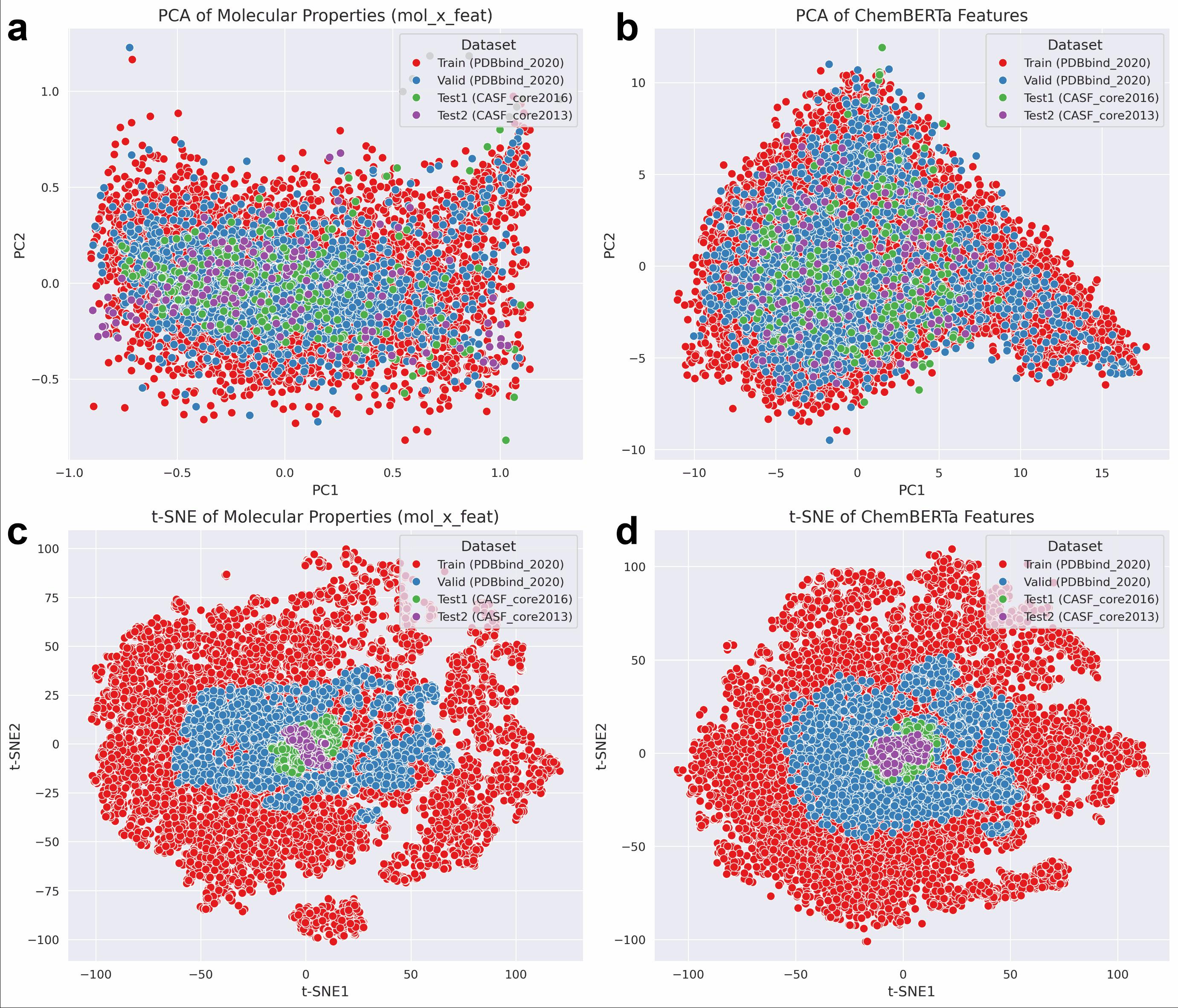

Supplement: btaf662_Supplementary_Data [file btaf662_supplementary_data.zip › figS2.png]

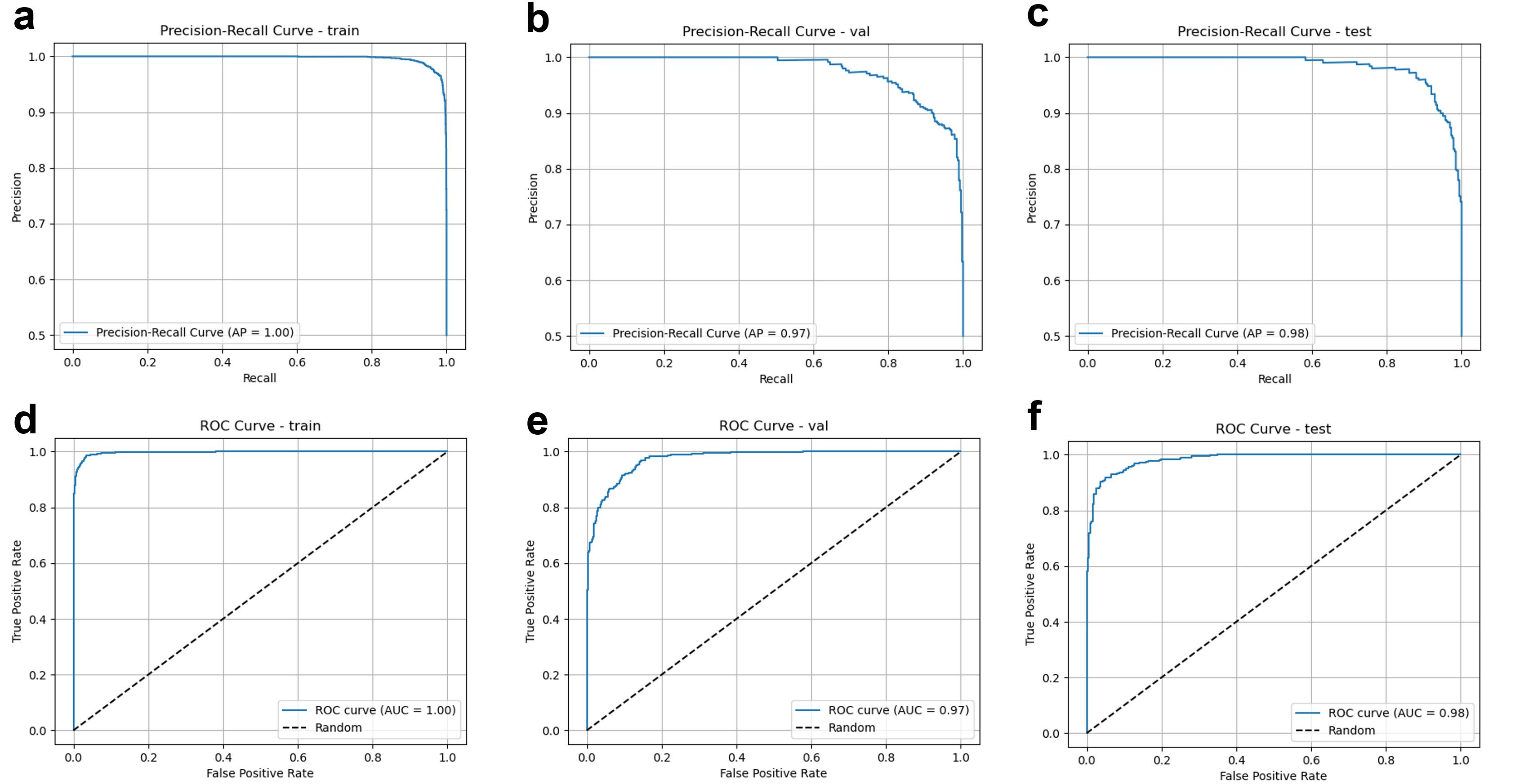

Supplement: btaf662_Supplementary_Data [file btaf662_supplementary_data.zip › figS3.png]

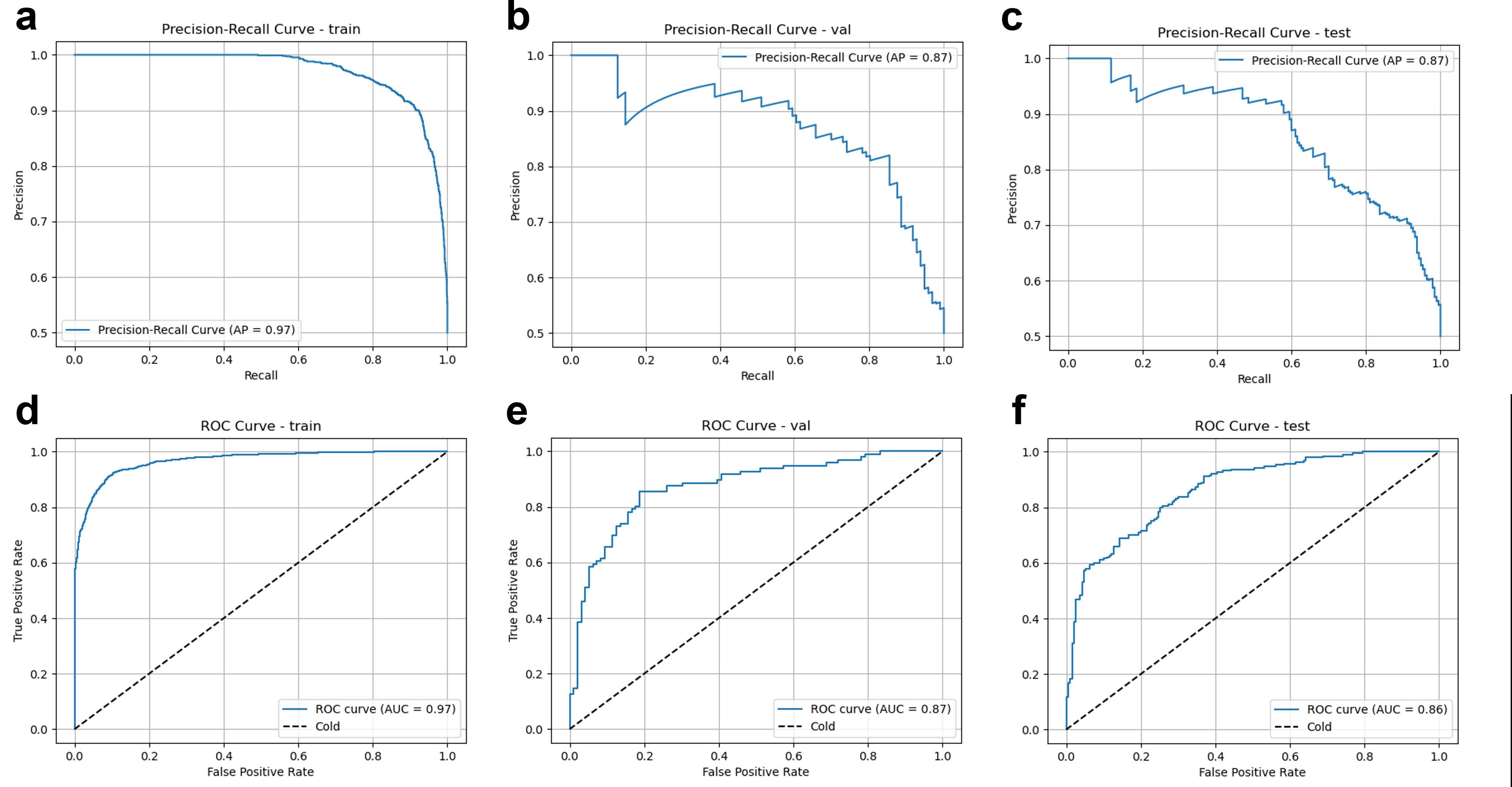

Supplement: btaf662_Supplementary_Data [file btaf662_supplementary_data.zip › figS4.png]

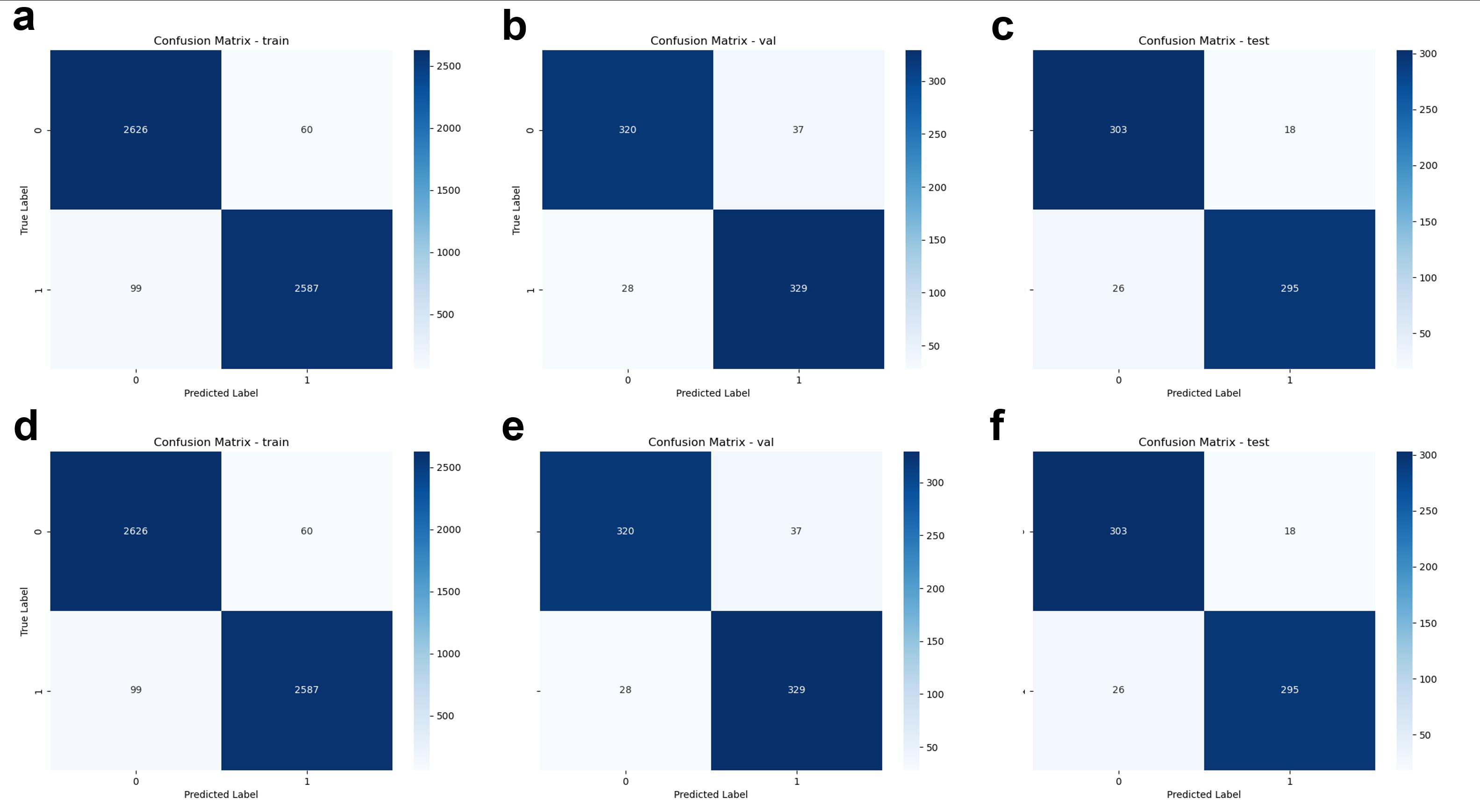

Supplement: btaf662_Supplementary_Data [file btaf662_supplementary_data.zip › figS5.png]

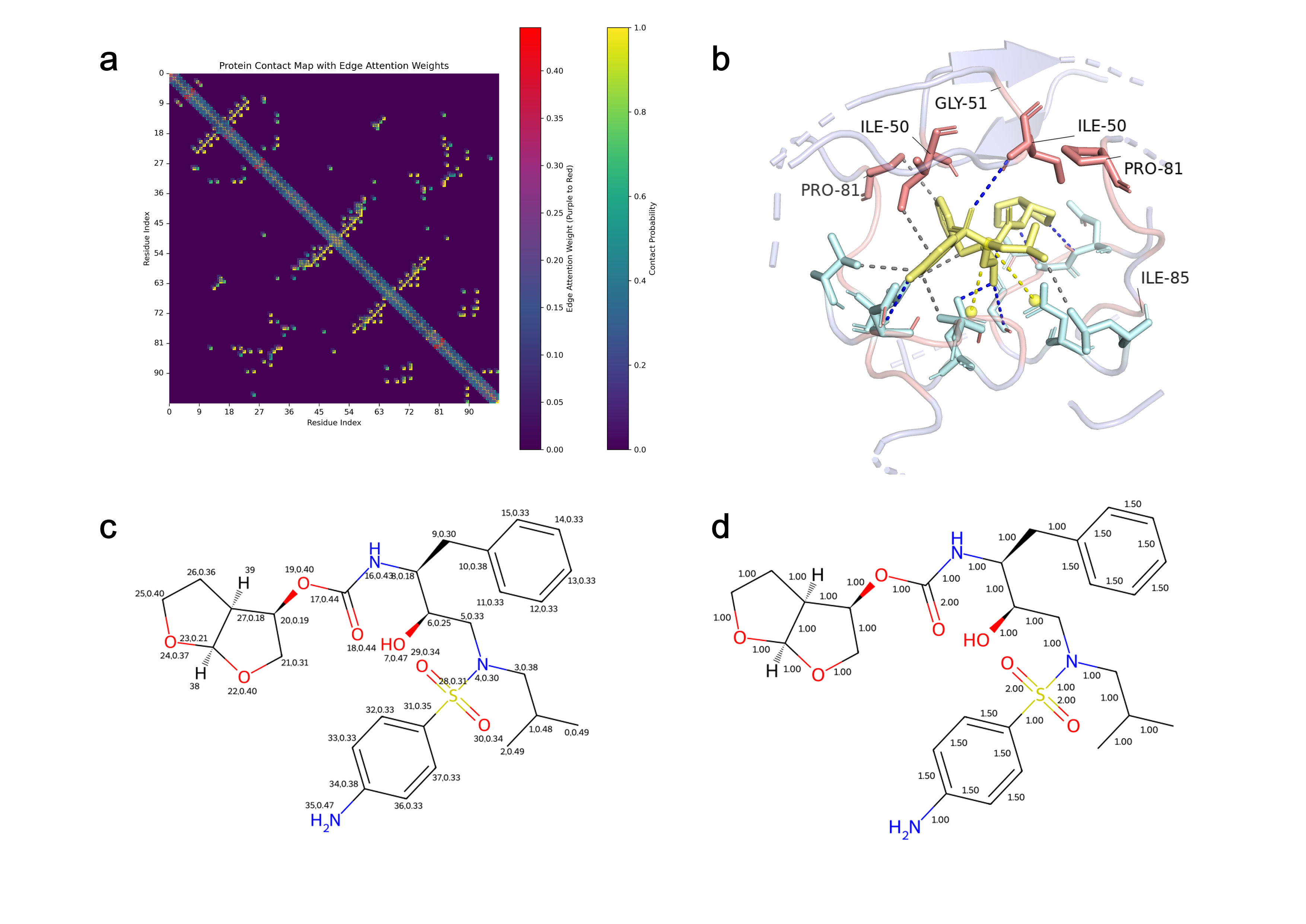

Supplement: btaf662_Supplementary_Data [file btaf662_supplementary_data.zip › figS6.png]

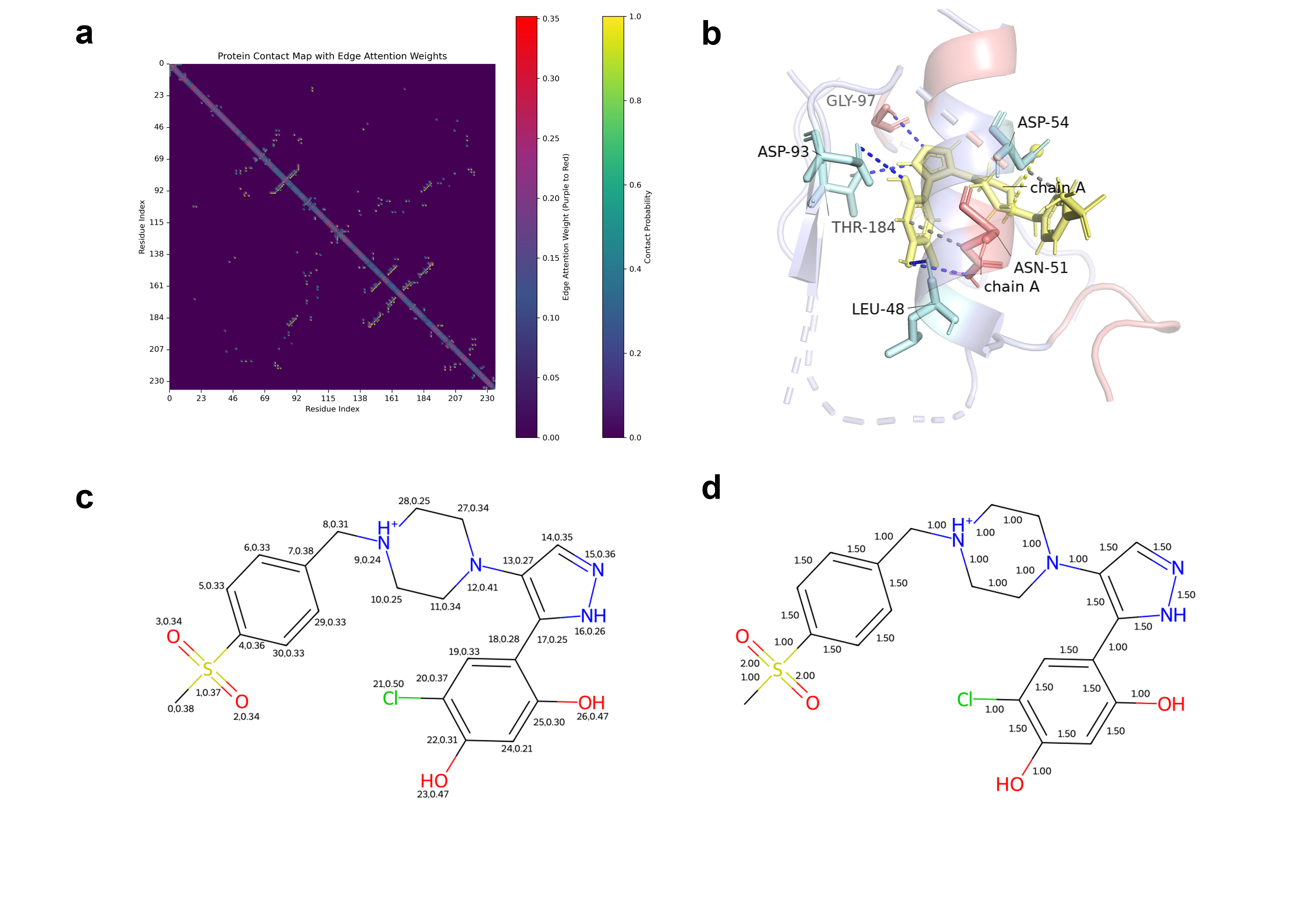

Supplement: btaf662_Supplementary_Data [file btaf662_supplementary_data.zip › figS7.png]
